# The use of glucose‐lowering medications for the treatment of type 2 diabetes mellitus during pregnancy in the United States

**DOI:** 10.1002/edm2.319

**Published:** 2021-12-24

**Authors:** Mollie E. Wood, Elisabetta Patorno, Krista F. Huybrechts, Brian T. Bateman, Kathryn J. Gray, Ellen W. Seely, Seanna Vine, Sonia Hernández‐Díaz

**Affiliations:** ^1^ Department of Epidemiology Harvard T.H. Chan School of Public Health Boston Massachusetts USA; ^2^ Division of Pharmacoepidemiology and Pharmacoeconomics Department of Medicine Brigham and Women's Hospital and Harvard Medical School Boston Massachusetts USA; ^3^ Department of Anesthesiology, Perioperative and Pain Medicine Brigham and Women’s Hospital Boston Massachusetts USA; ^4^ Division of Maternal‐Fetal Medicine Brigham and Women's Hospital and Harvard Medical School Boston Massachusetts USA; ^5^ Endocrinology, Diabetes and Hypertension Division Brigham and Women's Hospital and Harvard Medical School Boston Massachusetts USA; ^6^ Present address: Department of Anesthesiology, Perioperative and Pain Medicine Stanford University School of Medicine Stanford California USA

**Keywords:** drug utilization, pharmacoepidemiology, pharmacovigilance, pregnancy in diabetics

## Abstract

**Introduction:**

Some guidelines allow for the use of either insulin or noninsulin antidiabetic agents for gestational diabetes, but only insulin is recommended for pregnant women with preexisting type 2 diabetes mellitus (T2DM). We aimed to document treatment patterns in routine care for women with preexisting T2DM.

**Methods:**

We identified pregnancy cohorts within 2 US claims databases for publicly and privately insured individuals: the Medicaid Analytical eXtract (2000–2014) and OptumClinformatics (2004–2014). T2DM was classified with a validated algorithm using ICD‐9‐CM and CPT codes. We assessed medication usage over the years of the study, and changes in medication use before and after the beginning of pregnancy, using prescription fills as a proxy for the use of insulin, metformin, sulphonylureas and other noninsulin antidiabetic agents before pregnancy and during each trimester.

**Results:**

Among 12,631 women with T2DM, insulin use in pregnancy was stable over the study years (55%–60% in the 2nd trimester), but 2nd trimester use of metformin increased from <5% to 20%. Over the study period, 41% of women filled a prescription for metformin before pregnancy, 37% in the 1st trimester and 17% in the 2nd trimester. By the 2nd trimester, few women used sulphonylureas (11%) or other noninsulin antidiabetic agents (1%). Of the women on metformin only before pregnancy, 36% switched to insulin only by 2nd trimester, 11% added insulin and 16% continued on metformin only. Of the women on metformin and insulin before pregnancy, 61% switched to insulin only by 2nd trimester, 22% continued with metformin and insulin and <5% used only metformin.

**Conclusion:**

The use of insulin‐metformin combinations and other noninsulin antidiabetic drugs during pregnancy has increased. Safety studies for these medication regimens are needed.

## Graphical abstract

We examined prescription fills for glucose‐lowering medications among pregnant women with pregestational type 2 diabetes mellitus in the United States. The use of metformin during pregnancy increased over the study period, and transitions from noninsulin antidiabetic drugs to regimens including insulin often occurred after the start of pregnancy.

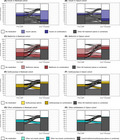

## 1 INTRODUCTION

Pre‐existing type 2 diabetes mellitus (T2DM) complicates 1% of US pregnancies annually, with substantially higher prevalence in medically underserved populations.^1^ Its prevalence is expected to increase in coming years given the obesity epidemic in the United States.^2^ T2DM is associated with an increased risk of poor maternal, foetal and neonatal outcomes, including pregnancy loss and stillbirth, preeclampsia, congenital malformations, macrosomia and birth injury.^3^, ^4^ Glycemic control is an important intermediary for these risks,^4^ highlighting the importance of preventing and treating hyperglycemia during pregnancy.

In nonpregnant women of reproductive age, T2DM is most often treated with lifestyle modifications; pharmacological treatment with antidiabetic agents is initiated if changes to diet and exercise are ineffective. Metformin is the preferred initial medication, and other agents, including insulin, can be added to achieve metabolic targets.^5^ However, whilst some guidelines allow for the use of noninsulin antidiabetic agents for gestational diabetes,^6^ the recommended treatment for preexisting T2DM during pregnancy is insulin.^6^ For women with T2DM considering pregnancy, current American Diabetes Association (ADA) and American College of Obstetrics and Gynecology (ACOG) guidelines recommend initiating insulin therapy as soon as possible, and ideally before pregnancy,^6^ although the use of metformin may be considered in rare cases.^7^

Nonetheless, the use of noninsulin antidiabetic agents by women with preexisting T2DM does occur in pregnancy.^8^, ^9^, ^10^ Recent studies have shown increases in the use of noninsulin antidiabetic medications during pregnancy, particularly metformin and glyburide.^8^, ^9^, ^11^ However, these studies were limited in their ability to describe medication use specifically in T2DM; classification of preexisting diabetes depended mostly^9^ or entirely^8^, ^11^ on filling prescriptions for antidiabetic medications before and/or during pregnancy and investigated populations that contained a mix of type 1, type 2 and gestational diabetes. Furthermore, although previous studies examined the prevalence of antidiabetic medication use in the pregnancy period overall, they did not describe within‐pregnancy longitudinal changes in treatment.

To address these limitations, we characterized prescription patterns and trends of antidiabetic medication use during pregnancy in two population‐based cohorts of publicly and privately insured pregnant women with pregestational T2DM.

## 2 MATERIALS AND METHODS

### 2.1 Data source and study population

Publicly insured pregnancies were identified from the US Medicaid Analytical eXtract for the period of 2000–2014. Construction of the Medicaid mother‐infant linked pregnancy cohort has been previously described in detail.^12^ For the privately insured cohort, we used OptumClinformatics (Optum) files from 2004 to 2015. Mother‐infant linkage in the Optum cohort was accomplished through a family identifier. Both Optum and Medicaid files contain diagnoses and procedure code emanating from inpatient stays and outpatient visits, as well as outpatient prescription fills. The date of the last menstrual period (LMP) was estimated based on the date of delivery and any codes for preterm birth, using a validated algorithm.^13^ Both cohorts consisted of women aged 12–55 years at the time of delivery who had pregnancies ending in live births, and who had continuous insurance coverage from 180 days before the LMP date to 30 days after delivery to ensure completeness of their pregnancy healthcare claims. Data supporting this study are not publicly available due to privacy/ethical restrictions.

### 2.2 Definition of pregestational type 2 diabetes mellitus

We previously developed and validated an algorithm based on *International Classification of Diseases*, *9th revision*, *Clinical Modification* (ICD‐9‐CM) diagnosis codes, *Current Procedural Terminology* (CPT) procedure codes and prescription fills, to classify pregestational diabetes in pregnant women (Table S1), with a positive predictive value (PPV) of 91.7% for any pregestational and 87.0% for T2DM.^14^ We modified the algorithm for this analysis to require ≥2 T2DM codes and 0 type 1 diabetes mellitus (T1DM) codes from 180 days before LMP through 90 days after LMP (PPV 100%; Table S2).

### 2.3 Definitions of pregnancy periods and medication use

We assessed diabetes medication use before pregnancy and during each trimester. The prepregnancy baseline period was defined as 90 days before LMP date to the day before the LMP date (LMP‐90 to LMP‐1). The first trimester was defined as LMP to day 90 of pregnancy (LMP to LMP + 90), the second trimester as days 91–180 after LMP (LMP + 91 to LMP + 180) and the third trimester as the period between day 181 and the delivery date (LMP + 181 to delivery).

We categorized antidiabetic medications or classes as insulin, metformin, sulphonylureas and other noninsulin antidiabetic agents. The latter category includes thiazolidinediones, alpha‐glucosidase inhibitors (AGI), sodium‐glucose cotransporter 2 inhibitors (SGLT2i), dipeptidyl peptidase‐4 inhibitors (DPP4i), glucagon‐like peptide‐1 receptor agonists (GLP1 RA), pramlintide and meglitinides (Table S3). Women were considered to have used the medication of interest if they filled a prescription in the relevant pregnancy period, and those who used a combination product were considered users of both products (eg a prescription for glyburide/metformin HCl would be counted in both the metformin and sulphonylurea groups).

We also defined 8 mutually exclusive exposure groups based on the most frequently observed treatment strategies: no pharmacological treatment, metformin only, insulin only, metformin and insulin, sulphonylureas only, sulphonylureas and metformin, sulphonylureas and other noninsulin antidiabetic drugs, and “other” that included any pregnancies whose pharmacological treatment was not described by the previous definitions.

### 2.4 Maternal characteristics

Maternal characteristics were coded using ICD‐9‐CM and CPT codes. Demographic characteristics were coded as the most recent value before the delivery date. Markers of diabetes severity, comorbid health conditions, laboratory test orders and concomitant medications were evaluated between LMP − 180 and LMP + 90. Healthcare utilization and preventive services were evaluated from LMP − 180 to LMP − 1 (Table S4).

### 2.5 Data analysis

We described secular trends in the use of specific antidiabetic medications or medication combinations in pregnancies with an estimated LMP occurring between 2000 and 2014 (Medicaid) and 2004 and 2014 (Optum), evaluated the prevalence of medication use during each prepregnancy and pregnancy period and examined within‐individual longitudinal patterns of medication use from prepregnancy to the second trimester, for all medications described above. Because this was a descriptive analysis, we did not conduct tests of statistical significance. Analyses were done separately for the Medicaid and Optum cohorts. Cell sizes <11 are suppressed to protect patient privacy.

We conducted several sensitivity analyses, which (1) repeated the main analysis using an alternate definition of T2DM that included women classified as either T2DM or pregestational diabetes not otherwise specified (NOS) by the algorithm (PPV 82.9%) (Table S2), and (2) stratified within‐pregnancy longitudinal analyses into pregnancies occurring before 2008 versus 2008 or later, to evaluate whether trends changed after the publication of a landmark randomized trial of metformin treatment for gestational diabetes.^15^

Cohort construction and descriptive analysis were carried out using SAS v9.4. Figures were created using the *ggplot2* and *ggalluvial* ^16^ packages in R/RStudio.

### 2.6 Ethics statement/informed consent

This study was approved by the Institutional Review Board at Mass General Brigham and the Harvard TH Chan School of Public Health. No informed consent was required.

## 3 RESULTS

### 3.1 Cohort characteristics

We identified 10,987 pregnancies from Medicaid and 1,644 from Optum (Table 1). The cohorts differed in several ways, including maternal age, with 47% of the Optum cohort being age 35 or older, compared to 26% of the Medicaid cohort. In addition, the Optum cohort received more referrals for nutrition counselling (12% vs. 5% in Medicaid) and had a higher proportion diagnosed with hyperlipidemia (39% vs. 19%), hypothyroidism (21% vs. 6%) and polycystic ovarian syndrome (PCOS) (13% vs. 3%); they also had more laboratory tests ordered. By contrast, the Medicaid cohort had a greater number of inpatient and outpatient encounters and filled more prescriptions for medications to treat hypertension (32% vs. 24% in Optum) as well as pain and psychiatric illnesses. Despite these differences, the cohorts were similar in terms of prevalence of diabetic complications, diabetes‐related comorbidities and other maternal health conditions.

**TABLE 1:** Characteristics of pregnant women with pregestational type 2 diabetes recorded in claims (N = 10,987 for Medicaid and N = 1,644 for Optum)

	Medicaid		Optum	
	N = 10,987		N = 1,644	
	N	%	N	%
Age				
24 and younger	2,103	19%	26	2%
25–29	2,952	27%	236	14%
30–34	3,170	29%	617	38%
35–39	2,034	19%	572	35%
40 and older	728	7%	193	12%
Laboratory tests ordered				
Haemoglobin A1c	7,583	69%	1,471	89%
Glucose	5,548	50%	726	44%
Metabolic panel	7,213	66%	1,298	79%
Lipid panel	4,490	41%	1,003	61%
Creatinine	849	8%	83	5%
Urine albumin‐to‐creatinine ratio	2,851	26%	606	37%
Number of laboratory tests (mean, SD)	4.9	5.3	4.3	6.4
Preventive services				
Glucose strips	1,018	9%	99	6%
Seasonal flu vaccine	866	8%	139	8%
Lifestyle risk factors				
Obesity	2,237	20%	434	26%
Nutritional counselling referral	600	5%	199	12%
Tobacco use	780	7%	58	4%
Diabetic complications				
Diabetic retinopathy	175	2%	29	2%
Other diabetes‐related ophthalmopathy	319	3%	30	2%
Diabetic neuropathy	146	1%	18	1%
Skin infections	1,068	10%	104	6%
Hyperglycemia	526	5%	111	7%
Hypoglycemia	211	2%	32	2%
Diabetic ketoacidosis	201	2%	24	1%
Diabetes complications NOS	872	7.9%	76	4.6%
Other diabetes‐related comorbidities				
Sleep apnoea	141	1%	27	2%
Polyuria/polydipsia	64	0.5%	14	0.1%
Polycystic ovarian syndrome	276	3%	207	13%
Hyperinsulinemia	37	0%	12	1%
Abnormal glucose	668	6%	161	10%
Glycosuria	44	0%	15	1%
Acanthosis nigricans	32	0%	14	1%
Maternal health conditions				
Hypertension	3,075	28%	506	31%
Hyperlipidemia	2,045	19%	633	39%
Other cardiometabolic conditions	1079	9.8%	152	9.2%
Asthma	192	2%	33	2%
Depression	1,589	14%	169	10%
Anxiety	1,033	9%	130	8%
Pneumonia	205	2%	17	1%
Oedema	208	2%	25	2%
Hypothyroidism	711	6%	339	21%
Hyperthyroidism	110	1%	34	2%
Chronic kidney disease or other renal conditions	211	1.9%	38	2.3%
Healthcare utilization				
Outpatient visits (mean, SD)	11.3	14.1	5.9	6.8
Emergency department visits (mean, SD)	2.8	1.2	2.0	0.3
Hospital admissions (mean, SD)	0.5	0.2	0.3	0.1
Days hospitalized (mean, SD)	2.6	0.7	1.6	0.3
Prescription medications				
Number of prescriptions (mean, SD)	5.5	8.0	4.0	5.2
Any antihypertensive drug	3,543	32%	390	24%
ACE inhibitors	2,222	20%	166	10%
Statins	1,825	17%	211	13%
Oral corticosteroids	987	9%	136	8%
NSAIDs	4,680	43%	233	14%
Opioids	5,211	47%	479	29%
Anticonvulsants	1,024	9%	53	3%
Antidepressants	2,458	22%	215	13%
Benzodiazepines	1,058	10%	113	7%
Thyroid replacement	596	5%	232	14%

Note

Additional variable information: age was coded as the most recent value before the delivery date. Markers of diabetes severity, comorbid health conditions, laboratory test orders and concomitant medications were evaluated between LMP − 180 and LMP + 90. Healthcare utilization and preventive services were evaluated from LMP − 180 to LMP − 1.

Abbreviations: ACE, angiotensin‐converting enzyme; LMP, last menstrual period; NOS, not otherwise specified; NSAID, nonsteroidal anti‐inflammatory drugs; SD, standard deviation.

### 3.2 Utilization prevalence of specific antidiabetics by trimester

**FIGURE 1:**
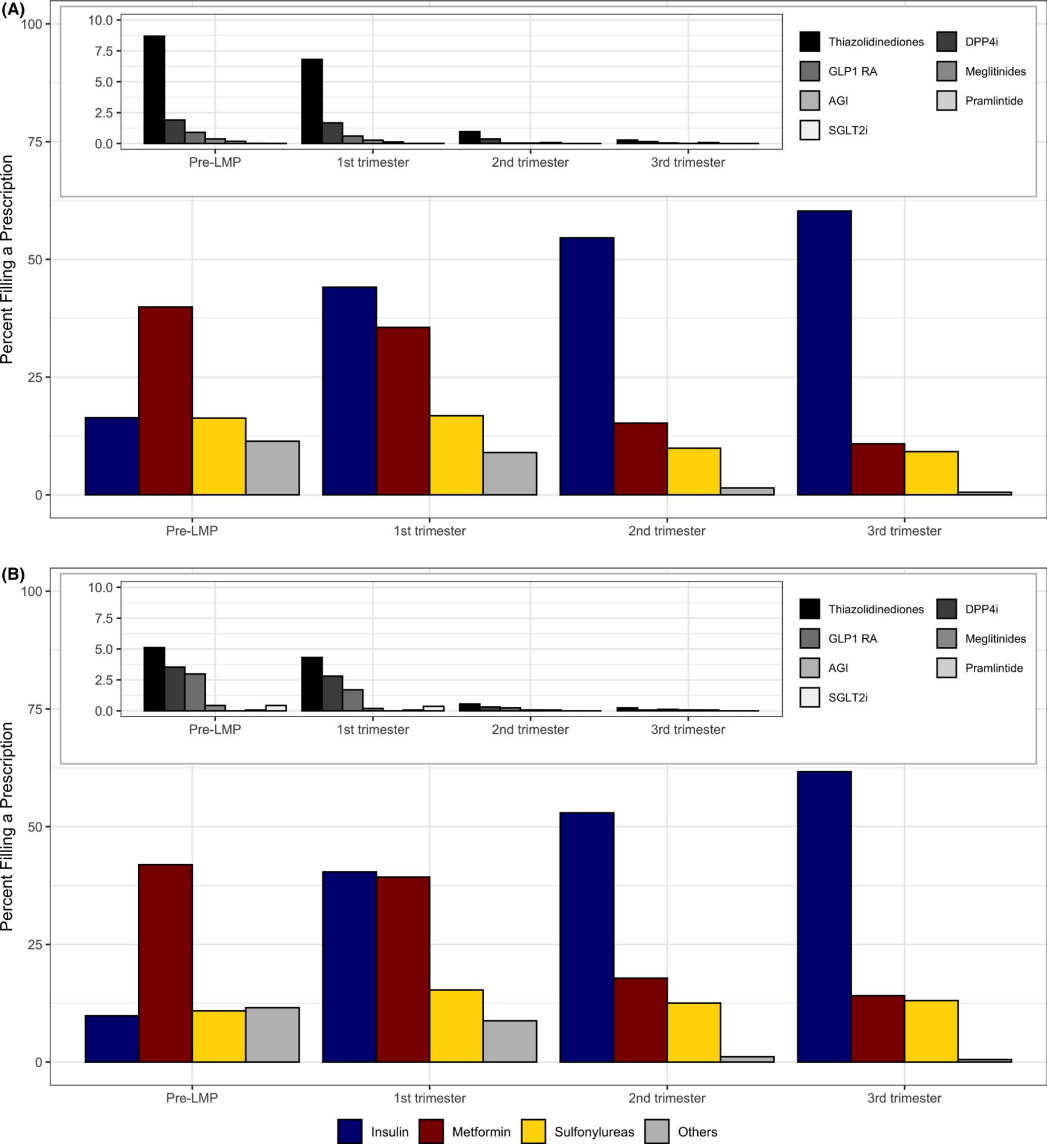
Proportion of women with type 2 diabetes using antidiabetic medications before and during pregnancy (A) among 10,987 Medicaid women (2000–2014) and (B) among 1,644 privately insured women (2004–2014). Inset plots show proportion of users for medications classified as “others” in the main plot. Pre‐LMP refers to the 90 days before the last menstrual period

Figure 1 shows the percentage of the sample who filled a prescription for each antidiabetic medication or class during each pregnancy period. In the 90 days before pregnancy, metformin was used by the largest proportion of women (40% in Medicaid and 42% in Optum); insulin and sulphonylureas were both used by 16% of women in Medicaid and 10%–11% of women in Optum; and other noninsulin antidiabetic medications were used by 10% and 12% of Medicaid and Optum participants respectively. Insulin dispensing increased during pregnancy, with corresponding decreases in other treatments: during the first trimester, 44% of Medicaid and 40% of Optum filled a prescription for insulin, whilst metformin prescription fills decreased to 36% and 39% respectively; 17% of Medicaid and 15% of Optum filled a prescription for a sulphonylurea; and 9% filled a prescription for other noninsulin antidiabetic medications. In the second trimester, insulin prescriptions were filled by 55% of Medicaid and 53% of Optum participants, versus 15% and 18% filling prescriptions for metformin, 10% of Medicaid and 13% of Optum filling prescriptions for sulphonylureas, and 1% filling prescriptions for other noninsulin antidiabetics. This pattern continued into the third trimester, with 60% of Medicaid and 62% of Optum filling prescriptions for insulin, 11% of Medicaid and 14% of Optum filling prescriptions for metformin, 9% of Medicaid and 13% of Optum filling prescriptions for a sulphonylurea, and 1% filled a prescription for other noninsulin antidiabetic medication.

### 3.3 Time trends for the prevalence of prescriptions for specific antidiabetics

**FIGURE 2:**
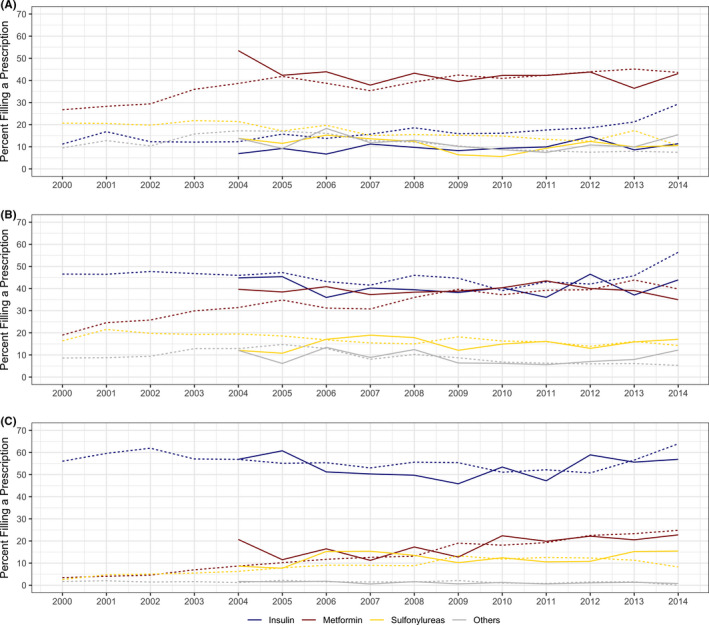
Secular trends in the use of antidiabetic medications by women with type 2 diabetes (A) before pregnancy, (B) during the first trimester and (C) during the second trimester. Dotted lines show proportions among 10,987 Medicaid women (2000–2014), and solid lines show proportions among 1,644 privately insured women (2004–2014). Medications may be used alone or in combination. “Others” includes thiazolidinediones, AGI, SGLT2i, DPP4i, GLP1 RA, pramlintide and meglitinides

The percentages of women with T2DM filling a prescription for insulin, metformin, sulphonylureas, and other noninsulin antidiabetic medications over time (2000–2014 for Medicaid, 2004–2014 for Optum) are shown in Figure 2. The use of insulin before pregnancy more than doubled over the study period (from 11% in 2000 to 29% in 2014 for Medicaid, and from 7% to 15% in Optum). Prepregnancy metformin use also increased for the Medicaid cohort (from 27% in 2000 to 44% in 2014) but remained relatively stable for the Optum cohort (between 37% and 43%, with the exception of 53% in 2004), whilst the use of sulphonylureas had modest decreases (Figure 2A). In the Medicaid cohort during the earlier years of observation, first trimester prescription fills for insulin were more than twice as common as metformin fills (11% filled a prescription for metformin vs. 27% for insulin in 2000), but metformin use increased more rapidly over the study period than insulin, and both medications were used by approximately 45% of women by 2014. In the Optum sample, first trimester fills for metformin and insulin were similar for the whole study period, with approximately 40% filling a prescription for either medication (Figure 2B). The use of metformin in the second trimester increased from less than 5% of pregnancies in 2000 to more than 20% in 2014 in the Medicaid cohort, with similar increases in the Optum cohort during the same period (11%–23%). Sulphonylureas also had a modest second trimester increase from less than 5% in 2000 to 13% in 2009 for Medicaid and from 8% in 2005 to 15% in 2007 for Optum and remaining steady in the following years (Figure 2C); most of the increase was due to glyburide. Compared to insulin, metformin and sulphonylureas, other noninsulin antidiabetic medications collectively accounted for a smaller proportion of treatment before pregnancy (up to 13% in Medicaid and 17% in Optum) and during pregnancy (in the second trimester <2% in both Medicaid and Optum), which was stable over the study period. Figure S1 gives additional detail.

### 3.4 Treatment strategies throughout pregnancy

Before pregnancy, the most common treatment strategy was metformin monotherapy (20% in Medicaid and 26% in Optum), followed by insulin monotherapy (9% in Medicaid and 5% in Optum). Insulin monotherapy was the most common treatment strategy by the second trimester (45% in Medicaid and 42% in Optum), with an increase in treatment strategies involving noninsulin medications over the study period (Figures S2 and S3). Among women filling a prescription for an antidiabetic treatment before pregnancy, most (73%) were dispensed metformin alone or in combination.

**FIGURE 3:**
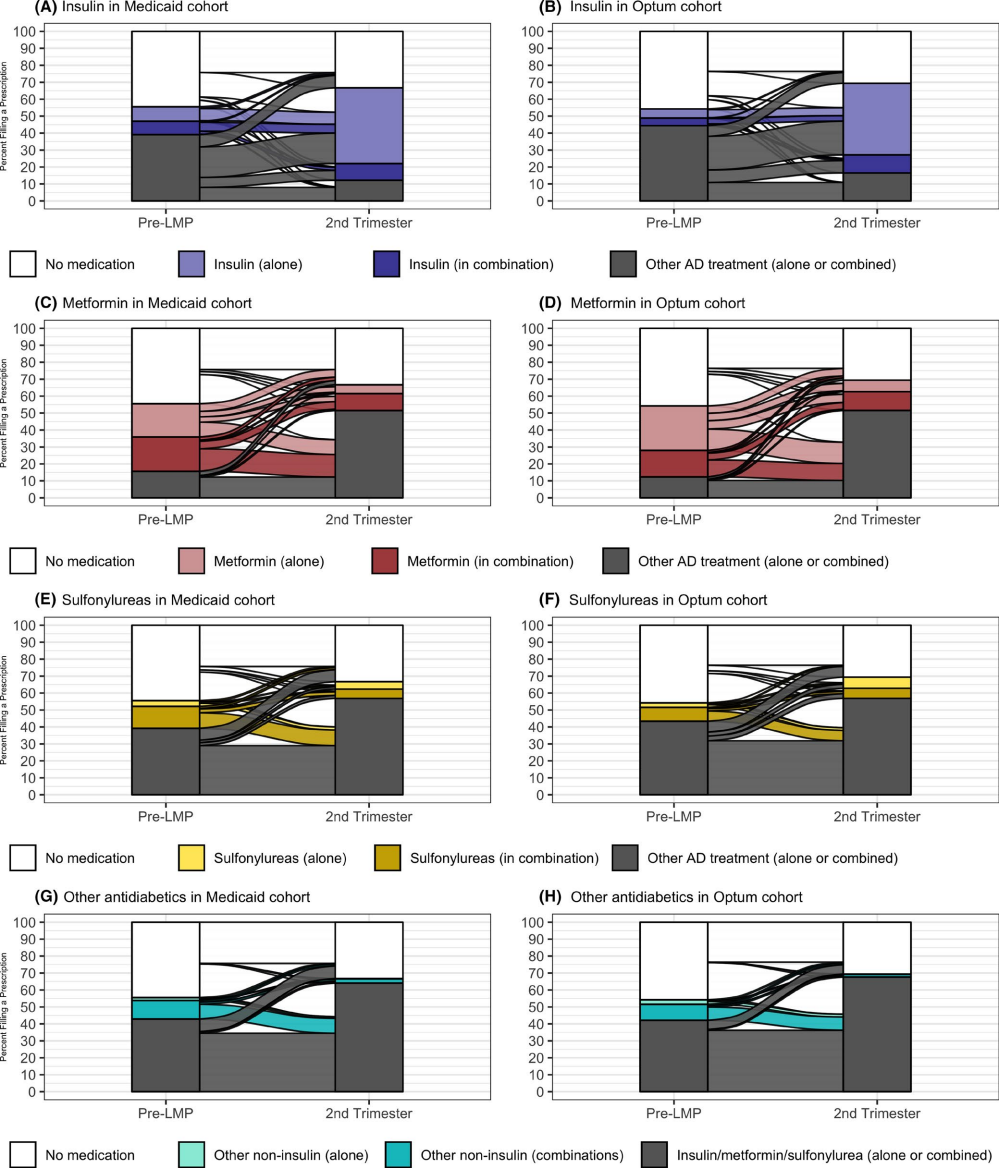
Longitudinal patterns in antidiabetic medications from before pregnancy to the second trimester. Horizontal flows show the proportion of users of a given drug, alone or in combination, as use changes. “Other AD treatment” is specific to each drug or class and references any diabetes medication not including that drug. “Other noninsulin” (panels G and H) that includes thiazolidinediones, AGI, SGLT2i, DPP4i, GLP1 RA and meglitinides. Panels A, C, E and G show proportions among 10,987 Medicaid women (2000–2014), and panels B, D, F and H show proportions among 1,644 privately insured women (2004–2014)

**FIGURE 4:**
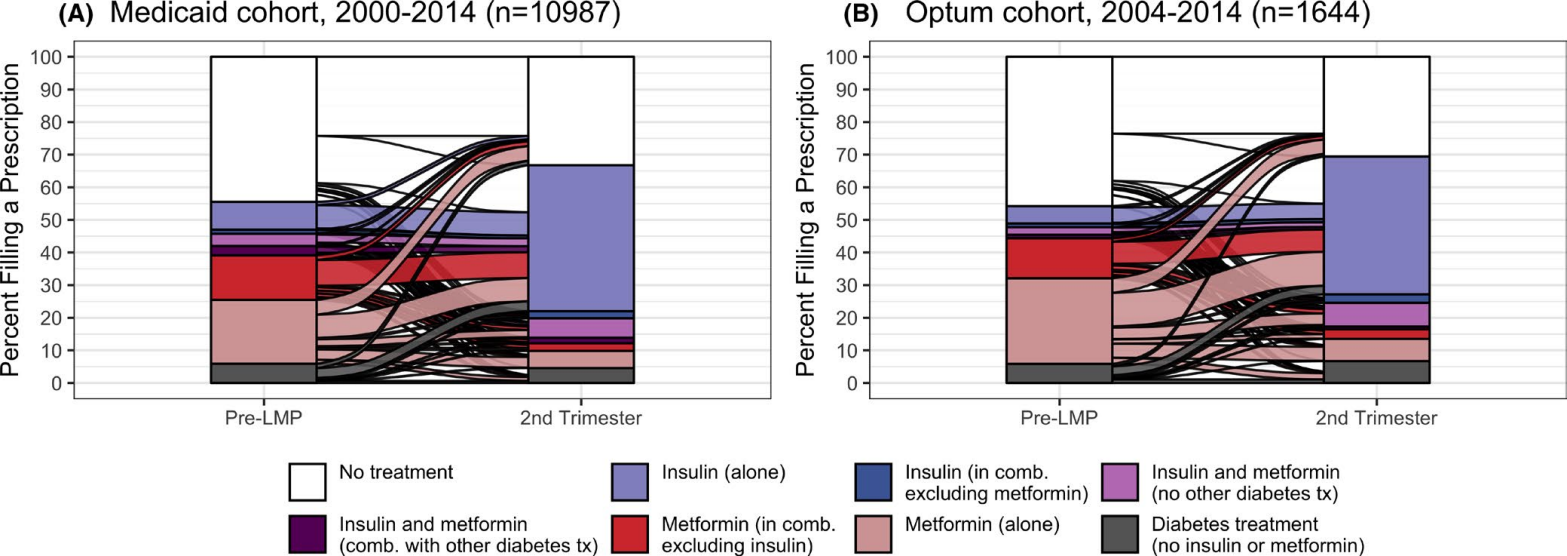
Longitudinal patterns in insulin and metformin treatment from before pregnancy to the second trimester. Horizontal flows show the proportion of users of a treatment strategy, alone or in combination, as use changes. “Diabetes treatment” references any diabetes medications, alone or in combination, not including insulin or metformin. Panel A shows proportions among 10,987 Medicaid women (2000–2014), and panel B shows proportions among 1,644 privately insured women (2004–2014)

Given that the first trimester seems to be a transitional period for antidiabetic treatment adjustment once a pregnancy is identified, we present trajectories of use from the prepregnancy period to the second trimester (Figures 3 and 4, with additional supporting information included in Tables S5 and S6). Five to seven percentage of T2DM women was on insulin monotherapy before pregnancy, and the majority continued on insulin monotherapy in the second trimester (Figure 3A and B). Among women dispensed metformin alone before pregnancy, 36%–39% (in Medicaid and Optum respectively) switched to insulin monotherapy and an additional 12%–14% augmented the treatment with insulin in the second trimester (Figure 3C and D, Figure 4). Among women on metformin combined with insulin before pregnancy, 65%–67% switched to insulin monotherapy in the second trimester and an additional 23%–27% continued on both metformin and insulin (Figure 4). Relative to patterns among women on metformin before pregnancy, fewer women with prepregnancy dispensations for sulphonylureas (Figure 3E and F) or other noninsulin antidiabetic drugs (Figure 3G and H) discontinued pharmacotherapy; instead, they switched or augmented treatment, most often with insulin. The supplemental material includes trajectories involving combinations of insulin with sulphonylureas (Figure S4, Table S7). Approximately 25% of women with T2DM were not prescribed a medication to treat diabetes before pregnancy and continued without pharmacotherapy through the second trimester. An additional 20%–22% filled no prescriptions for antidiabetic medications before pregnancy and initiated pharmacotherapy by the second trimester, mostly with insulin monotherapy (65%–71%; Figure 4).

### 3.5 Sensitivity analyses

Stratified analyses before 2008 vs. 2008 or later (Figures S5‐S10) showed that, after 2008, fewer women switched to insulin monotherapy in the second trimester (Figure S5), and more women switched to a treatment regime that included metformin (Figure S6). Sensitivity analyses examining utilization with a more sensitive definition of T2DM showed patterns of treatment that were consistent with the main analysis (Figures S11‐S18).

## 4 COMMENT

### 4.1 Principal findings

In a large cohort of publicly and privately insured US women with T2DM, metformin was the most frequently dispensed antidiabetic medication in the 90 days before pregnancy. The prescription for metformin declined throughout pregnancy, and insulin monotherapy was the most prevalent treatment by the second trimester. However, the use of metformin during pregnancy more than doubled between 2000 and 2014, whilst insulin use was stable. Treatment combinations of insulin with other noninsulin antidiabetic medications, predominantly metformin, also increased over time. Women on metformin monotherapy before pregnancy often switched to, or added, insulin, whilst those on insulin monotherapy rarely added metformin. The smaller number of women on the combination of metformin and insulin before pregnancy tended to stay on the combination. Sulphonylurea prescriptions were less common but remained stable both throughout pregnancy and over time. The use of antidiabetic medications other than insulin, metformin and sulphonylureas before pregnancy was low, and most women switched to insulin early in pregnancy.

Our results are consistent with other recent studies describing increases in the use of noninsulin antidiabetic medications in pregnancy.^8^, ^9^, ^10^, ^11^ We observed that metformin was the most commonly prescribed antidiabetic medication before pregnancy, which differs from studies in which insulin was more prevalent^8^, ^9^; this discrepancy is likely due to differences in the definition of pregestational diabetes, used in other studies which allowed for inclusion of women with T1DM.

In addition, nearly 1 in 4 women in our sample were classified as having pregestational T2DM but filled no prescriptions for antidiabetic medications. These results are similar to a study that used diagnosis codes, rather than filled prescriptions, to classify pregestational diabetes type,^10^ and are likely an indication of management through diet and exercise. The percentage of women managed through lifestyle in our sample was stable over time, so even if some of the women not treated with antidiabetic medications are not “true” T2DM cases, we would not expect our conclusions about relative medication prevalence to change.

There are several possible explanations for the observed increase in metformin use during pregnancy. First, as many pregnancies are unplanned,^17^ changes to prepregnancy medication regimens often occur during pregnancy. Metformin and newer noninsulin drugs are established treatments in the nonpregnant T2DM population, and late recognition of pregnancy or lack of preconception counselling may lead to continuation of these medications into pregnancy. Consistent with this hypothesis, transitions to guideline‐recommended treatment^6^, ^7^ are not occurring before pregnancy for many women in our study, which may result in first trimester foetal exposure to medications with unknown safety profiles and/or suboptimal glycemic control in early pregnancy. Second, because women with T2DM often have insulin resistance that worsens during pregnancy, they may require high doses of insulin to achieve euglycemia.^6^ The possibility that metformin in combination with insulin may help pregnant women with T2DM achieve glycemic control without high doses of insulin has been embraced by many clinicians and is the subject of two randomized trials.^18^, ^19^ The MiTy trial evaluated the addition of metformin to a standard insulin regimen among pregnant women with T2DM enrolled between 6 and 20 weeks of gestation and found no differences in neonatal mortality or serious morbidity, as well as a significant benefit in the metformin group for glycemic control, lower insulin requirements, less gestational weight gain and fewer caesarean births; infants in the metformin group weighed less, which resulted in both fewer infants born extremely large for gestational age but also more infants born small for gestational age,^19^ indicating a need for long‐term surveillance. In fact, understanding the effects of prenatal exposure to noninsulin antidiabetic drugs on foetal development and long‐term outcomes in offspring remains an urgent research gap.^20^ As of 2014, the newer noninsulin antidiabetic drugs increasingly used by the adult population had been largely avoided by the pregnant women in the United States. However, because a substantial fraction of women do not change medication regimens until the first or second trimester, future studies should evaluate the efficacy and safety of noninsulin antidiabetics in early pregnacy.

### 4.2 Strengths and limitations

Our study has several important limitations. First, we used the date of a prescription fill to indicate medication use during a specific period, which may result in both overestimation and underestimation. A woman may fill a prescription for a medication but may discontinue its use after learning she is pregnant; on the other hand, a woman may have filled a prescription in an earlier period and have a supply overlapping with a later period, such that she was truly exposed in both periods but would only be classified as exposed in the earlier period. Reliance on prescription fills could also produce the appearance of concomitant medication use, and fail to correctly identify treatment switching. Second, our study ends in 2014, which limits our ability to assess newer noninsulin antidiabetic treatments and recent changes in clinical practice. Third, misclassification of T2DM is possible and may differ within specific treatment patterns (eg metformin users may include more non‐T2DM women incorrectly classified as T2DM because metformin is used for other indications than diabetes, whilst insulin users with T2DM may be misclassified as T1DM). However, because our validated algorithm required the presence of multiple specific diagnostic codes to classify individuals as T2DM,^14^ we expect this type of misclassification to be infrequent. Finally, we required women to have been continuously enrolled in their health insurance plan from 180 days before pregnancy start through 30 days post delivery, which may have selected a sample whose characteristics differ from the underlying population of pregnant women with T2DM in terms of wealth and/or disability.

These limitations are balanced by several strengths. We conducted our study in a large cohort that included both publicly and privately insured women in the United States and used a validated algorithm to identify preexisting T2DM based on ICD‐9 codes and prescription fills before and early in pregnancy. The results were almost identical in the publicly and privately insured populations. We also used data visualization methods to describe longitudinal trajectories of medication use to gain a better understanding of the transition of treatment after pregnancy onset. This study highlights important future research directions for the evaluation of pharmacological management of T2DM during pregnancy by identifying the most common treatment strategies.

### 4.3 Conclusions

In a large population of US women with T2DM between 2000 and 2014, metformin was the most frequently used antidiabetic medication before pregnancy, whereas insulin monotherapy became the most prevalent treatment by the second trimester. The use of metformin during pregnancy more than doubled between 2000 and 2014, and treatment combinations of insulin with other antidiabetic medications, predominantly metformin, also increased over time. Given the increasing use of noninsulin antidiabetic medications in early pregnancy, safety studies are needed.

## CONFLICT OF INTEREST

EP is an investigator of a research grant to the Brigham and Women's Hospital from Boehringer Ingelheim, not related to the topic of the submitted work. KFH reports being an investigator on research grants to Brigham and Women's Hospital from Eli Lilly and GlaxoSmithKline for unrelated studies. BTB received research grants to Brigham and Women's Hospital from Eli Lilly, Baxalta and Pacira for unrelated studies; personal fees from Aetion and from Alosa Foundation outside the submitted work; and served on an expert panel for a postpartum haemorrhage quality improvement project that was conducted by the Association of Women's Health, Obstetric and Neonatal Nurses and funded by a grant from Merck for Mothers. KJG reports nonfinancial support from Illumina Inc., personal fees from Quest Diagnostics, personal fees from BillionToOne and personal fees from Aetion Inc. outside the submitted work. SHD reports being an investigator on grants to her institution from Takeda for unrelated studies; personal fees from UCB and Roche outside the submitted work; and having served as an epidemiologist with the North America AED pregnancy registry, which is funded by multiple companies. MEW, SV and EWS report no conflicts of interest.

## AUTHOR CONTRIBUTIONS

**Mollie E. Wood:** Conceptualization (equal); Data curation (equal); Formal analysis (equal); Methodology (lead); Visualization (lead); Writing – original draft (lead); Writing – review & editing (equal). **Elisabetta Patorno:** Conceptualization (equal); Project administration (equal); Supervision (supporting); Writing – review & editing (equal). **Krista F. Huybrechts:** Conceptualization (supporting); Project administration (supporting); Supervision (supporting); Writing – review & editing (equal). **Brian T Bateman:** Writing – review & editing (equal). **Kathryn Gray:** Writing – review & editing (equal). **Ellen W. Seely:** Writing – review & editing (equal). **Seanna Vine:** Data curation (lead); Formal analysis (lead). **Sonia Hernández‐Díaz:** Conceptualization (lead); Funding acquisition (lead); Project administration (lead); Supervision (lead); Writing – review & editing (equal).

## Supporting information

Supplementary Material

## Data Availability

Data supporting this study are not publicly available due to privacy/ethical restrictions.
