# Supplementary material for: The use of glucose‐lowering medications for the treatment of type 2 diabetes mellitus during pregnancy in the United States
Source: Endocrinol Diabetes Metab. 2021 Dec 24;5(2):e00319. doi: 10.1002/edm2.319 (PMC8917861; doi:10.1002/edm2.319)
Supplement: Supplementary file 1 — Supplementary Material [file EDM2-5-e00319-s001.docx]

SUPPLEMENTAL MATERIAL

**Supplemental Table S1**. ICD-9-CM codes used for diabetes classification

| **ICD-9-CM** | **Definition** |
| --- | --- |
|  |  |
| 250.xx | **Diabetes mellitus NOS** (non-specific).  ICD-9: (forth digit indicates mention of complication).  If no number in fifth digit, consider Unspecified pregestational diabetes |
| 250.x1, 250.x3 | **Type 1 pregestational diabetes** |
| 250.x0, 250.x2 | **Type 2 pregestational diabetes** |
| 648.8x | **Gestational diabetes**  ICD-9: Abnormal glucose tolerance complicating pregnancy (considered gestational diabetes)  ICD-10: Gestational diabetes mellitus and abnormal glucose complicating… |
| 648.0x | **Pregestational NOS**  Codes for “diabetes mellitus complicating pregnancy childbirth or the puerperium” (consider Pregestational but UNSPECIFIC, i.e. not otherwise specified (NOS) |

**Supplemental Table S2**. Positive predictive values for algorithm to identify pregestational diabetes: sensitivity analysis for (a) original algorithm, (b) main definition requiring 2 or more type 2 codes and 0 type 1 codes, and (c) alternate definition combining algorithm type 2 and NOS definitions

|  | No. records reviewed^1^ | No. records confirmed | Diagnoses from EMR review^2^ | PPV (95% CI) |
| --- | --- | --- | --- | --- |
| *(a) original algorithm* | | | | |
| Pregestational diabetes (any)^3^ | 49 | 45 | 3 gestational diabetes  1 insufficient information | 91.8 (82.0 to 96.5) |
| Type 2 | 23 | 20 | 2 gestational diabetes  1 insufficient information | 87.0 (68.4 to 95.4) |
| Pregestational NOS | 12 | NA^4^ | 11 pregestational  (2 type 1, 9 type 2)  1 gestational | NA^4^ |
| *(b) definition of type 2 modified to require 2 specific type 2 codes and 0 type 1 codes* | | | | |
| Type 2 | 6 | 6 | All confirmed by chart review | 100 |
| *(c) definition of type 2 modified to include type 2 and NOS from original algorithm* | | | | |
| Type 2 | 35 | 29 | 3 gestational diabetes  2 type 1 diabetes  1 insufficient information | 82.9 (69.7 to 91.1) |

Abbreviations: CI: confidence interval; EMR: electronic medical record; No.: number; PPV: positive predictive value

1. Number excludes observations with missing electronic medical record for the index pregnancy

2. For observations misclassified by the algorithm, correct classifications are provided

3. Includes records identified as type 1 or type 2, plus records classified as pregestational diabetes NOS

4. Not calculated/reported due to lack of diagnostic standard

**Supplemental Table S3.** Antidiabetic medications

| **Group Name** | **Compound** | **Generics Included** |
| --- | --- | --- |
| *Insulin* | | |
| Insulins  OR | Insulin | Insulin Aspart Protamine/ Insulin Aspart; Insulin Aspart, Recombinant; Insulin Aspart/ Insulin Aspart Protamine; Insulin Detemir; Insuline Glargine, Recombinant; Insulin Glulisine; Insulin Human Isophane; Insulan Human Isophane/ Insulan Human Regular; Insulin Human Regular; Insulin Human Zinc (LEnte); Insulin Lispro Protamine/ Insulin Lispro; Insulin Lispro, Recombinant; Insulin Lispro/ Insulin Lispro Protamine |
| These CPT codes | V58.67 (ICD9) | Long-term use of insulin |
| *Metformin* | | |
| Biguanides | Metformin | Alogliptin Benzoate/ Metformin HCl; Canagliflozin/ Metformin HCl; Glipizide/ Metformin HCl; Glyburide/ Metformin HCl; Linagliptin/ Metformin HCl; Metformin HCl; Metformin HCl/ Pioglitazone HCl; Metformin HCl/ Rosiglitazone Maleate; Metformin HCl/ Saxagliptin HCl; Metformin HCl/ Sitagliptin Phosphate |
| *Sulfonylureas* | | |
| Sulfonylureas | Glimepiride | Glimepiride; Glimepiride/Pioglitazone Hydrochloride |
| Sulfonylureas | Glipizide | Glipizide; Glipizide/ Metformin Hydrochloride |
| Sulfonylureas | Glyburide | Glyburide; Glyburide, Micronized; Glyburide/ Metformin Hydrochloride |
| Sulfonylureas | Acetohexamide | Acetohexamide |
| Sulfonylureas | Chlorprompamide | Chlorpropamide |
| Sulfonylureas | Tolazamide | Tolazamide |
| Sulfonylureas | Tolbutamide | Tolbutamide |
| *Other antidiabetic medications* | | |
| Pramlintide | Pramlintide | Pramlintide Acetate |
| Thiazolidinediones | Pioglitazone | Glimepiride/ Pioglitazone HCl; Metformin HCl/ Pioglitazone HCl; Pioglitazone HCl |
| Thiazolidinediones | Rosiglitazone | Metformin HCl/Rosiglitazone Maleat; Rosiglitazone Maleate |
| AGI | Acarbose | Acarbose |
| AGI | Miglitol | Miglitol |
| SGLT2i | Dapagliflozin | Dapagliflozin; Dapagliflozin/Metformin HCl; dapagliflozin/saxagliptin |
| SGLT2i | Empagliflozin | Empagliflozin; Empagliflozin/Metformin HCl; Empagliflozin/Linagliptin |
| SGLT2i | Canagliflozin | Canagliflozin; Canagliflozin/ Metformin HCl |
| SGLT2i | Ertugliflozin | Ertugliflozin |
| DPP4i | Alogliptin | Alogliptin Benzoate; Alogliptin Benozoate/ Metformin HCl; Alogliptin/Pioglitazone |
| DPP4i | Linagliptin | Linagliptin; Linagliptin/ Metformin HCl |
| DPP4i | Saxagliptin | Metformin HCl/ Saxagliptin HCl; Saxagliptin HCl |
| DPP4i | Sitagliptin | Metformin HCl/ Sitagliptin Phosphate; Simvastatin/ Sitagliptin Phosphate; Sitagliptin Phosphate |
| GLP1 RA | Exenatide | Exenatide |
| GLP1 RA | Liraglutide | Liraglutide (exclude “Saxenda”, a formulation only used for obesity) |
| GLP1 RA | Albiglutide | Albiglutide |
| GLP1 RA | Dulaglutide | Dulaglutide |
| GLP1 RA | Semaglutide | Semaglutide |
| Meglitinides | Nateglinide | Nateglinide |
| Meglitinides | Repaglinide | Repaglinide |

**Supplemental Table S4.** Prescription drugs, ICD-9-CM, HCPCP, CPT codes used to define characteristics for individuals included in the study cohorts

| Hb A1c | CPT code: 83036 |
| --- | --- |
| Glucose | CPT codes: 82947, 82948, 82962 |
| Metabolic panel | CPT codes: 80048, 80050, 80053, 80054 |
| Lipid panel | CPT codes: 84478, 80061, 83721, 83701, 83704, 80061, 83718, 80061, 82465, 80061 |
| Creatinine | CPT code: 84520 |
| UACR | CPT codes: 82043, 82570 |
| Seasonal flu vaccine | ICD-9-CM: V04.81, V06.6  HCPCS: G9141, G9142, 90470, 90663, Q2033, Q2034, Q2035, Q2036, Q2037, Q2038, Q2039, G0008  CPT codes: 90470,90663,Q2033,Q2034,Q2035,Q2036,Q2037,Q2038,Q2039,G0008,V04.81,V06.6, 90630, 90653, 90654, 90655, 90656, 90657, 90658, 90660, 90661, 90662, 90664, 90666, 90667, 90668, 90672, 90673, 90674, 90682, 90685, 90686, 90687, 90688  National drug codes omitted due to very long list; available from the authors upon request |
| Obesity | ICD-9-CM: 278.00, 278.01, 278.03, 649.1x, V85.3x, V85.4x, V65.3, 278.02, 539.xx, 649.2x, 43.82, 43.89, 44.31, 44.38, 44.39, 44.68, 44.69, 44.93, 44.95, 44.98, 45.51, 45.91  CPT codes: 43644, 43645, 43659, 43770, 43842, 43843, 43844, 43845, 43846, 43847, 43999, S2082, 97802, 97803  Generic drug names: lorcaserin, phentermine/ topiramate, bupropion/ naltrexone, phentermine, diethylpropion, phendimetrazine, benzphetamine, sibutramine, dexfenfluramine, fenfluramine |
| Nutritional counseling referral | ICD-9-CM: V65.3  CPT codes: 97803, 97803 |
| Tobacco use | ICD-9-CM: 305.1x, 649.0x, 989.84, V15.82  CPT codes: 99406, 99407 |
| Diabetic retinopathy | ICD-9-CM: 362.0x |
| Other diabetes-related ophthalmopathy | ICD-9-CM: 250.5x, 366.41, 365.44, 362.83 |
| Diabetic neuropathy | ICD-9-CM: 250.4x, 583.81 |
| Skin Infections | ICD-9-CM: 680.xxx-686.xx |
| Hyperglycemia | ICD-9-CM: 790.29 |
| Hypoglycemia | ICD-9-CM: 251.1x, 251.2, 962.3 |
| Diabetes complications NOS | ICD-9-CM: 250.8x, 250.9x |
| Diabetic ketoacidosis | ICD-9-CM: 250.1x, 250.2x, 250.3x |
| Sleep apnea | ICD-9-CM: 327.23 |
| Pneumonia | ICD-9-CM: 480.xx, 481, 482.xx, 483.xx 484.xx, 485, 486, 487.0, 507.xx |
| Edema | ICD-9-CM: 782.3x |
| Polyuria/polydipsia | ICD-9-CM: 788.42, 783.5 |
| Hypothyroidism | ICD-9-CM: 243, 244.xx |
| Hyperthyroidism | ICD-9-CM: 242.1x, 242.3x, 242.9x |
| Polycystic ovarian syndrome | ICD-9-CM: 256.4 |
| Hyperinsulinemia | ICD-9-CM: 251.1 |
| Abnormal glucose | ICD-9-CM: 790.2x |
| Glycosuria | ICD-9-CM: 791.5 |
| Acanthosis nigricans | ICD-9-CM: 701.2 |
| Hypertension | ICD-9-CM: 401.xx-405.xx, 642.0x-642.2x, 642.7x, 642.9x |
| Hyperlipidemia | ICD-9-CM: 272.0, 272.2, 272.4 |
| Other cardiometabolic conditions | ICD-9-CM: 410.xx, 411.xx, 412.xx, 413.xx, 414.xx, 430.xx , 431.xx, 432.xx, 433.xx, 434.xx, 435.xx, 436.xx, 437.xx, 438.xx, 428.xx, 398.91, 402.01, 402.11, 402.91, 404.01, 404.11, 404.91, 404.01, 404.13, 404.91, 427.xx, 440.2x, 440.3x, 440.4, 443.9, 390.xx-392.xx, 393.xx-398.xx, 415.xx-417.xx, 420.xx-429.xx, 440.0, 440.1, 440.8, 441.xx-449.xx, 451.xx-459.xx |
| Chronic kidney disease or other renal conditions | ICD-9-CM: 585.3 585.4 585.5 585.6 582.xx 583.xx 585.1 585.2 585.9 586.xx 587.xx, 642.1x, 250.4x, 250.4, 250.41, 250.42, 250.43, 403.xx, 404.xx, 572.4x, 580.xx, 584.xx, 580.0x, 580.4x, 580.89, 580.9x, 582.4x, 642.1x, 791.2x, 791.3x |
| Asthma | ICD-9-CM: 493.0x, 493.1x, 493.8x, 493.0x |
| Depression | ICD-9-CM: 293.83, 296.2x. 296.3x, 298.0x, 300.4x, 309.0x, 309.1x, 309.28, 311.xx |
| Anxiety | ICD-9-CM: 293.84, 300.0x, 300.2x, 300.3x, 309.24, 308.0x, 309.81, 313.0x |
| Prescription Medications |  |
| Any antihypertensive drug | benazepril hcl, captopril, enalapril maleate, fosinopril sodium, fosenopril sodium, fosfenopril, fosinopril diacid, lisinopril, ramipril, trandolapril, moexipril hcl, perindopril arginine/perindopril erbumine, quinapril hcl, azilsartan kamedoxomil, candesartan cilexetil, eprosartan mesylate, irbesartan, losartan potassium, olmesartan medoxomil, telmisartan, valsartan, acebutolol, atenolol, betaxolol, bisoprolol, carteolol, carvedilol, esmolol, labetalol, metoprolol tartrate, metoprolol succinate, propranolol, penbutolol, pindolol, nadolol, nebivolol, sotalol, timolol, benzthiazide, bendroflumethiazide, chlorothiazide, chlorothiazide sodium, hydrochlorothiazide, methyclothiazide, polythiazide, hydroflumethiazide, indapamide, metolazone, quinethazone, trichlormethiazide, furosemide, bumetanide, torsemide, ethacrynic acid, amiloride, eplerenone, spironolactone, triamterene, diltiazem, mibefradil, verapamil, amlodipine, clevidipine, bepridil, felodipine, isradipine, nicardipine, nifedipine, nimodipine, nisoldipine, nitroglycerin, isosorbide dinitrate, isosorbide mononitrate, ranolazine, doxazosin, eplerenone, prazosin, terazosin, clonidine, guanabenz, guanadrel, guanethidine, guanfacine, hydralazine, methyldopa, metyrosine, reserpine, minoxidil, aliskiren, benazepril hcl, captopril, enalapril maleate, fosinopril sodium, fosenopril sodium, fosfenopril, fosinopril diacid, lisinopril, ramipril, trandolapril, moexipril hcl, perindopril arginine/perindopril erbumine, quinapril hcl, azilsartan kamedoxomil, candesartan cilexetil, eprosartan mesylate, irbesartan, losartan potassium, olmesartan medoxomil, telmisartan, valsartan, acebutolol, atenolol, betaxolol, bisoprolol, carteolol, carvedilol, esmolol, labetalol, metoprolol tartrate, metoprolol succinate, propranolol, penbutolol, pindolol, nadolol, nebivolol, sotalol, timolol, benzthiazide, bendroflumethiazide, chlorothiazide, chlorothiazide sodium, hydrochlorothiazide, methyclothiazide, polythiazide, hydroflumethiazide, indapamide, metolazone, quinethazone, trichlormethiazide, furosemide, bumetanide, torsemide, ethacrynic acid, amiloride, eplerenone, spironolactone, triamterene, diltiazem, mibefradil, verapamil, amlodipine, clevidipine, bepridil, felodipine, isradipine, nicardipine, nifedipine, nimodipine, nisoldipine, nitroglycerin, isosorbide dinitrate, isosorbide mononitrate, ranolazine, doxazosin, eplerenone, prazosin, terazosin, clonidine, guanabenz, guanadrel, guanethidine, guanfacine, hydralazine, methyldopa, metyrosine, reserpine, minoxidil, aliskiren, digoxin |
| ACE inhibitors | benazepril hcl, captopril, enalapril maleate, fosinopril sodium, fosenopril sodium, fosfenopril, fosinopril diacid, lisinopril, ramipril, trandolapril, moexipril hcl, perindopril arginine/perindopril erbumine, quinapril hcl |
| Statins | atorvastatin calcium, fluvastatin sodium, lovastatin, pitavastatin calcium, pravastatin sodium, rosuvastatin calcium, simvastatin, niacin, nicotinic acid, niacinamide, fenofibrate, gemfibrozil, cholestyramine, colesevelam, colestipol, ezetimibe alone |
| Oral corticosteroids | prednisone, hydrocortisone, cortisone acetate, prednisolone, methylprednisolone, dexamethasone, triamcinolone, betamethasone |
| NSAIDs | aspirin, celecoxib, diclofenac, diflunisal, etodolac, fenoprofen, flurbirofen, ibuprofen, indomethacin, ketroprofen, ketorolac, mefenamic acid, meloxicam, nabumetone, naproxen, oxaprozin, piroxicam, sulindac, tolmetin |
| Opioids | buprenorphine, butorphanol, codeine, fentanyl, hydrocodone, hydromorphone, levorphanol, meperidine, methadone, morphine, oxycodone, oxymorphone, pentazocine, propoxyphene, tapentadol, tramadol |
| Anticonvulsants | carbamazepine, eslicarbazepine, ethotoin, ethosuximide, ezogabine, felbamate, fosphenytoin, gabapentin, lacosamide, lamotrigine, levetiracetam, mephenytoin, mephobarbital, methsuximide, oxcarbazepine, perampanel, phenobarbital, phenytoin, pregabalin, primidone, rufinamide, tiagabine, topiramate, valproatedivalproex, valproic acid, valproate sodium, vigabatrin, zonisamide |
| Antidepressants | citalopram, escitalopram, fluoxetine, fluvoxamine, paroxetine, sertraline, desvenlafaxine, levomilnacipran, venlafaxine, amitriptyline, amoxapine, clomipramine, desipramine, doxepin, imipramine, maprotiline, nortriptyline, protriptyline, trimipramine, isocarboxazid, phenelzine, tranylcypropine, bupropion, mirtazapine, nefazodone, trazodone, vilazodone, vortioxetine |
| Benzodiazepines | alprazolam, chlordiazepoxide, clonazepam, clorazepate, diazepam, estazolam, flurazepam, halazepam, lorazepam, midazolam, oxazepam, prazepam, quazepam, temazepam, triazolam |
| Thyroid replacement | levothyroxine sodium, liothyronine sodium, liotrix |

**Supplemental Table S5.** Number and percent of individuals following treatment trajectories involving insulin, metformin, sulfonylureas, and other antidiabetic medications from pre-pregnancy to second trimester.

| **Treatment Trajectory** | | **MAX** | | **Optum** | |
| --- | --- | --- | --- | --- | --- |
| **Pre-pregnancy** | **2nd Trimester** | **N** | **Percent** | **N** | **Percent** |
| **Insulin** |  |  |  |  |  |
| No medication | No medication | 2668 | 24.3 | 388 | 23.6 |
| No medication | Insulin (alone) | 1584 | 14.4 | 237 | 14.4 |
| No medication | Other AD treatment (alone or combined) | 426 | 3.9 | 91 | 5.5 |
| No medication | Insulin (in combination) | 208 | 1.9 | 36 | 2.2 |
| Insulin (alone) | No medication | 113 | 1.0 | <10* |  |
| Insulin (alone) | Insulin (alone) | 780 | 7.1 | 78 | 4.7 |
| Insulin (alone) | Other AD treatment (alone or combined) | <10 |  | <10 |  |
| Insulin (alone) | Insulin (in combination) | 41 | 0.4 | <10 |  |
| Other AD treatment (alone or combined) | No medication | 805 | 7.3 | 104 | 6.3 |
| Other AD treatment (alone or combined) | Insulin (alone) | 1979 | 18.0 | 326 | 19.8 |
| Other AD treatment (alone or combined) | Other AD treatment (alone or combined) | 877 | 8.0 | 179 | 10.9 |
| Other AD treatment (alone or combined) | Insulin (in combination) | 640 | 5.8 | 121 | 7.4 |
| Insulin (in combination) | No medication | 67 | 0.6 | <10 |  |
| Insulin (in combination) | Insulin (alone) | 571 | 5.2 | 53 | 3.2 |
| Insulin (in combination) | Other AD treatment (alone or combined) | 30 | 0.3 | <10 |  |
| Insulin (in combination) | Insulin (in combination) | 192 | 1.7 | 16 | 1.0 |
| **Metformin** |  |  |  |  |  |
| No medication | No medication | 2668 | 24.3 | 388 | 23.6 |
| No medication | Metformin (alone) | 138 | 1.3 | 31 | 1.9 |
| No medication | Other AD treatment (alone or combined) | 1896 | 17.3 | 308 | 18.7 |
| No medication | Metformin (in combination) | 184 | 1.7 | 25 | 1.5 |
| Metformin (alone) | No medication | 499 | 4.5 | 73 | 4.4 |
| Metformin (alone) | Metformin (alone) | 356 | 3.2 | 71 | 4.3 |
| Metformin (alone) | Other AD treatment (alone or combined) | 957 | 8.7 | 207 | 12.6 |
| Metformin (alone) | Metformin (in combination) | 340 | 3.1 | 81 | 4.9 |
| Other AD treatment (alone or combined) | No medication | 272 | 2.5 | 22 | 1.3 |
| Other AD treatment (alone or combined) | Metformin (alone) | 15 | 0.1 | <10 |  |
| Other AD treatment (alone or combined) | Other AD treatment (alone or combined) | 1348 | 12.3 | 169 | 10.3 |
| Other AD treatment (alone or combined) | Metformin (in combination) | 82 | 0.7 | 11 | 0.7 |
| Metformin (in combination) | No medication | 214 | 1.9 | 20 | 1.2 |
| Metformin (in combination) | Metformin (alone) | 67 | 0.6 | <10 |  |
| Metformin (in combination) | Other AD treatment (alone or combined) | 1461 | 13.3 | 164 | 10.0 |
| Metformin (in combination) | Metformin (in combination) | 490 | 4.5 | 65 | 4.0 |
| **Sulfonylureas** |  |  |  |  |  |
| No medication | No medication | 2668 | 24.3 | 388 | 23.6 |
| No medication | Sulfonylureas (alone) | 231 | 2.1 | 54 | 3.3 |
| No medication | Other AD treatment (alone or combined) | 1850 | 16.8 | 285 | 17.3 |
| No medication | Sulfonylureas (in combination) | 137 | 1.2 | 25 | 1.5 |
| Sulfonylureas (alone) | No medication | 84 | 0.8 | <10 |  |
| Sulfonylureas (alone) | Sulfonylureas (alone) | 43 | 0.4 | <10 |  |
| Sulfonylureas (alone) | Other AD treatment (alone or combined) | 202 | 1.8 | 26 | 1.6 |
| Sulfonylureas (alone) | Sulfonylureas (in combination) | 42 | 0.4 |  |  |
| Other AD treatment (alone or combined) | No medication | 771 | 7.0 | 105 | 6.4 |
| Other AD treatment (alone or combined) | Sulfonylureas (alone) | 164 | 1.5 | 39 | 2.4 |
| Other AD treatment (alone or combined) | Other AD treatment (alone or combined) | 3184 | 29.0 | 524 | 31.9 |
| Other AD treatment (alone or combined) | Sulfonylureas (in combination) | 191 | 1.7 | 45 | 2.7 |
| Sulfonylureas (in combination) | No medication | 130 | 1.2 | <10 |  |
| Sulfonylureas (in combination) | Sulfonylureas (alone) | 44 | 0.4 | <10 |  |
| Sulfonylureas (in combination) | Other AD treatment (alone or combined) | 1009 | 9.2 | 100 | 6.1 |
| Sulfonylureas (in combination) | Sulfonylureas (in combination) | 237 | 2.2 | 22 | 1.3 |
| **Others** |  |  |  |  |  |
| No medication | No medication | 2668 | 24.3 | 388 | 23.6 |
| No medication | Other non-insulin (alone) | <10 |  | <10 |  |
| No medication | Insulin/metformin/sulfonylureas (alone or combined) | 2178 | 19.8 | 361 | 22.0 |
| No medication | Other non-insulin (in combination) | 36 | 0.3 | <10 |  |
| Other non-insulin (alone) | No medication | 55 | 0.5 | <10 |  |
| Other non-insulin (alone) | Other non-insulin (alone) | <10 |  | <10 |  |
| Other non-insulin (alone) | Insulin/metformin/sulfonylureas (alone or combined) | 111 | 1.0 | 27 | 1.6 |
| Other non-insulin (alone) | Other non-insulin (in combination) | 11 | 0.1 | <10 |  |
| Insulin/metformin/sulfonylureas (alone or combined) | No medication | 815 | 7.4 | 90 | 5.5 |
| Insulin/metformin/sulfonylureas (alone or combined) | Other non-insulin (alone) | <10 |  | <10 |  |
| Insulin/metformin/sulfonylureas (alone or combined) | Insulin/metformin/sulfonylureas (alone or combined) | 3791 | 34.5 | 596 | 36.3 |
| Insulin/metformin/sulfonylureas (alone or combined) | Other non-insulin (in combination) | 105 | 1.0 | <10 |  |
| Other non-insulin (in combination) | No medication | 115 | 1.0 | 15 | 0.9 |
| Other non-insulin (in combination) | Other non-insulin (alone) | <10 |  | <10 |  |
| Other non-insulin (in combination) | Insulin/metformin/sulfonylureas (alone or combined) | 961 | 8.7 | 128 | 7.8 |
| Other non-insulin (in combination) | Other non-insulin (in combination) | 126 | 1.1 | 12 | 0.7 |

*Cell counts of 10 or less are suppressed to protect patient privacy

**Supplemental Table S6.** Number and percent of individuals following treatment trajectories involving metformin and/or insulin from pre-pregnancy to second trimester.

| **Treatment Trajectory** | | **MAX** | | **Optum** | |
| --- | --- | --- | --- | --- | --- |
| **Pre-pregnancy** | **2nd Trimester** | **N** | **Percent** | **N** | **Percent** |
| No medication | No medication | 2668 | 24.3 | 388 | 23.6 |
| No medication | Insulin (alone) | 1584 | 14.4 | 237 | 14.4 |
| No medication | Metformin (alone) | 138 | 1.3 | 31 | 1.9 |
| No medication | Insulin and metformin  (no other diabetes tx) | 113 | 1.0 | 16 | 1.0 |
| No medication | Diabetes treatment  (no insulin or metformin) | 237 | 2.2 | 54 | 3.3 |
| No medication | Insulin (in comb.  excluding metformin) | 75 | 0.7 | 17 | 1.0 |
| No medication | Metformin (in comb.  excluding insulin) | 51 | 0.5 | <10 |  |
| No medication | Insulin and metformin  (comb. with other diabetes tx) | 20 | 0.2 | <10 |  |
| Insulin (alone) | No medication | 113 | 1.0 | <10 |  |
| Insulin (alone) | Insulin (alone) | 780 | 7.1 | 78 | 4.7 |
| Insulin (alone) | Metformin (alone) | <10* |  | <10 |  |
| Insulin (alone) | Insulin and metformin  (no other diabetes tx) | 31 | 0.3 | <10 |  |
| Insulin (alone) | Diabetes treatment  (no insulin or metformin) | <10 |  | <10 |  |
| Insulin (alone) | Insulin (in comb.  excluding metformin) | <10 |  | <10 |  |
| Insulin (alone) | Metformin (in comb.  excluding insulin) | <10 |  | <10 |  |
| Insulin (alone) | Insulin and metformin  (comb. with other diabetes tx) | <10 |  | <10 |  |
| Metformin (alone) | No medication | 499 | 4.5 | 73 | 4.4 |
| Metformin (alone) | Insulin (alone) | 780 | 7.1 | 170 | 10.3 |
| Metformin (alone) | Metformin (alone) | 356 | 3.2 | 71 | 4.3 |
| Metformin (alone) | Insulin and metformin  (no other diabetes tx) | 240 | 2.2 | 58 | 3.5 |
| Metformin (alone) | Diabetes treatment  (no insulin or metformin) | 131 | 1.2 | 31 | 1.9 |
| Metformin (alone) | Insulin (in comb.  excluding metformin) | 46 | 0.4 | <10 |  |
| Metformin (alone) | Metformin (in comb.  excluding insulin) | 75 | 0.7 | 22 | 1.3 |
| Metformin (alone) | Insulin and metformin  (comb. with other diabetes tx) | 25 | 0.2 | <10 |  |
| Insulin and metformin  (no other diabetes tx) | No medication | 35 | 0.3 | <10 |  |
| Insulin and metformin  (no other diabetes tx) | Insulin (alone) | 267 | 2.4 | 23 | 1.4 |
| Insulin and metformin  (no other diabetes tx) | Metformin (alone) | 14 | 0.1 | <10 |  |
| Insulin and metformin  (no other diabetes tx) | Insulin and metformin  (no other diabetes tx) | 88 | 0.8 | 12 | 0.7 |
| Insulin and metformin  (no other diabetes tx) | Diabetes treatment  (no insulin or metformin) | <10 |  | <10 |  |
| Insulin and metformin  (no other diabetes tx) | Insulin (in comb.  excluding metformin) | <10 |  | <10 |  |
| Insulin and metformin  (no other diabetes tx) | Metformin (in comb.  excluding insulin) | <10 |  | <10 |  |
| Insulin and metformin  (no other diabetes tx) | Insulin and metformin  (comb. with other diabetes tx) | <10 |  | <10 |  |
| Diabetes treatment  (no insulin or metformin) | No medication | 148 | 1.3 | 13 | 0.8 |
| Diabetes treatment  (no insulin or metformin) | Insulin (alone) | 335 | 3.0 | 44 | 2.7 |
| Diabetes treatment  (no insulin or metformin) | Metformin (alone) | 12 | 0.1 | <10 |  |
| Diabetes treatment  (no insulin or metformin) | Insulin and metformin  (no other diabetes tx) | 16 | 0.1 | <10 |  |
| Diabetes treatment  (no insulin or metformin) | Diabetes treatment  (no insulin or metformin) | 71 | 0.6 | 18 | 1.1 |
| Diabetes treatment  (no insulin or metformin) | Insulin (in comb.  excluding metformin) | 41 | 0.4 | 11 | 0.7 |
| Diabetes treatment  (no insulin or metformin) | Metformin (in comb.  excluding insulin) | 17 | 0.2 | <10 |  |
| Diabetes treatment  (no insulin or metformin) | Insulin and metformin  (comb. with other diabetes tx) | <10 |  | <10 |  |
| Insulin (in comb.  excluding metformin) | No medication | 11 | 0.1 | <10 |  |
| Insulin (in comb.  excluding metformin) | Insulin (alone) | 96 | 0.9 | 16 | 1.0 |
| Insulin (in comb.  excluding metformin) | Metformin (alone) | <10 |  | <10 |  |
| Insulin (in comb.  excluding metformin) | Insulin and metformin  (no other diabetes tx) | <10 |  | <10 |  |
| Insulin (in comb.  excluding metformin) | Diabetes treatment  (no insulin or metformin) | <10 |  | <10 |  |
| Insulin (in comb.  excluding metformin) | Insulin (in comb.  excluding metformin) | <10 |  | <10 |  |
| Insulin (in comb.  excluding metformin) | Metformin (in comb.  excluding insulin) | <10 |  | <10 |  |
| Insulin (in comb.  excluding metformin) | Insulin and metformin  (comb. with other diabetes tx) | <10 |  | <10 |  |
| Metformin (in comb.  excluding insulin) | No medication | 158 | 1.4 | 18 | 1.1 |
| Metformin (in comb.  excluding insulin) | Insulin (alone) | 864 | 7.9 | 112 | 6.8 |
| Metformin (in comb.  excluding insulin) | Metformin (alone) | 51 | 0.5 | <10 |  |
| Metformin (in comb.  excluding insulin) | Insulin and metformin  (no other diabetes tx) | 119 | 1.1 | 22 | 1.3 |
| Metformin (in comb.  excluding insulin) | Diabetes treatment  (no insulin or metformin) | 52 | 0.5 | <10 |  |
| Metformin (in comb.  excluding insulin) | Insulin (in comb.  excluding metformin) | 49 | 0.4 | <10 |  |
| Metformin (in comb.  excluding insulin) | Metformin (in comb.  excluding insulin) | 112 | 1.0 | 18 | 1.1 |
| Metformin (in comb.  excluding insulin) | Insulin and metformin  (comb. with other diabetes tx) | 96 | 0.9 | <10 |  |
| Insulin and metformin  (comb. with other diabetes tx) | No medication | 21 | 0.2 | <10 |  |
| Insulin and metformin  (comb. with other diabetes tx) | Insulin (alone) | 208 | 1.9 | 14 | 0.9 |
| Insulin and metformin  (comb. with other diabetes tx) | Metformin (alone) | <10 |  | <10 |  |
| Insulin and metformin  (comb. with other diabetes tx) | Insulin and metformin  (no other diabetes tx) | 39 | 0.4 | <10 |  |
| Insulin and metformin  (comb. with other diabetes tx) | Diabetes treatment  (no insulin or metformin) | <10 |  | <10 |  |
| Insulin and metformin  (comb. with other diabetes tx) | Insulin (in comb.  excluding metformin) | <10 |  | <10 |  |
| Insulin and metformin  (comb. with other diabetes tx) | Metformin (in comb.  excluding insulin) | <10 |  | <10 |  |
| Insulin and metformin  (comb. with other diabetes tx) | Insulin and metformin  (comb. with other diabetes tx) | 31 | 0.3 | <10 |  |

*Cell counts of 10 or less are suppressed to protect patient privacy

**Supplemental Table S7.** Number and percent of individuals following treatment trajectories involving sulfonylureas and/or insulin from pre-pregnancy to second trimester.

| **Treatment Trajectory** | | **MAX** | | **Optum** | |
| --- | --- | --- | --- | --- | --- |
| **Pre-pregnancy** | **2nd Trimester** | **N** | **Percent** | **N** | **Percent** |
| No medication | No medication | 2668 | 24.3 | 388 | 23.6 |
| No medication | Insulin (alone) | 1584 | 14.4 | 237 | 14.4 |
| No medication | Sulfonylureas (alone) | 231 | 2.1 | 54 | 3.3 |
| No medication | Insulin and sulfonylureas  (no other diabetes tx) | 69 | 0.6 | 17 | 1.0 |
| No medication | Diabetes treatment  (no insulin or sulfonylureas) | 145 | 1.3 | 31 | 1.9 |
| No medication | Insulin (in comb.  excluding sulfonylureas) | 121 | 1.1 | 17 | 1.0 |
| No medication | Sulfonylureas (in comb.  excluding insulin) | 50 | 0.5 | <10* |  |
| No medication | Insulin and sulfonylureas  (comb. with other diabetes tx) | 18 | 0.2 | <10 |  |
| Insulin (alone) | No medication | 113 | 1.0 | <10 |  |
| Insulin (alone) | Insulin (alone) | 780 | 7.1 | 78 | 4.7 |
| Insulin (alone) | Sulfonylureas (alone) | <10 |  | <10 |  |
| Insulin (alone) | Insulin and sulfonylureas  (no other diabetes tx) | <10 |  | <10 |  |
| Insulin (alone) | Diabetes treatment  (no insulin or sulfonylureas) | <10 |  | <10 |  |
| Insulin (alone) | Insulin (in comb.  excluding sulfonylureas) | 31 | 0.3 | <10 |  |
| Insulin (alone) | Sulfonylureas (in comb.  excluding insulin) | <10 |  | <10 |  |
| Insulin (alone) | Insulin and sulfonylureas  (comb. with other diabetes tx) | <10 |  | <10 |  |
| Sulfonylureas (alone) | No medication | 84 | 0.8 | <10 |  |
| Sulfonylureas (alone) | Insulin (alone) | 191 | 1.7 | 22 | 1.3 |
| Sulfonylureas (alone) | Sulfonylureas (alone) | 43 | 0.4 | <10 |  |
| Sulfonylureas (alone) | Insulin and sulfonylureas  (no other diabetes tx) | 27 | 0.2 | <10 |  |
| Sulfonylureas (alone) | Diabetes treatment  (no insulin or sulfonylureas) | <10 |  | <10 |  |
| Sulfonylureas (alone) | Insulin (in comb.  excluding sulfonylureas) | <10 |  | <10 |  |
| Sulfonylureas (alone) | Sulfonylureas (in comb.  excluding insulin) | <10 |  | <10 |  |
| Sulfonylureas (alone) | Insulin and sulfonylureas  (comb. with other diabetes tx) | <10 |  | <10 |  |
| Insulin and sulfonylureas  (no other diabetes tx) | No medication | <10 |  | <10 |  |
| Insulin and sulfonylureas  (no other diabetes tx) | Insulin (alone) | 40 | 0.4 | <10 |  |
| Insulin and sulfonylureas  (no other diabetes tx) | Sulfonylureas (alone) | <10 |  | <10 |  |
| Insulin and sulfonylureas  (no other diabetes tx) | Insulin and sulfonylureas  (no other diabetes tx) | <10 |  | <10 |  |
| Insulin and sulfonylureas  (no other diabetes tx) | Diabetes treatment  (no insulin or sulfonylureas) | <10 |  | <10 |  |
| Insulin and sulfonylureas  (no other diabetes tx) | Insulin (in comb.  excluding sulfonylureas) | <10 |  | <10 |  |
| Insulin and sulfonylureas  (no other diabetes tx) | Sulfonylureas (in comb.  excluding insulin) | <10 |  | <10 |  |
| Insulin and sulfonylureas  (no other diabetes tx) | Insulin and sulfonylureas  (comb. with other diabetes tx) | <10 |  | <10 |  |
| Diabetes treatment  (no insulin or sulfonylureas) | No medication | 615 | 5.6 | 96 | 5.8 |
| Diabetes treatment  (no insulin or sulfonylureas) | Insulin (alone) | 1103 | 10.0 | 234 | 14.2 |
| Diabetes treatment  (no insulin or sulfonylureas) | Sulfonylureas (alone) | 155 | 1.4 | 39 | 2.4 |
| Diabetes treatment  (no insulin or sulfonylureas) | Insulin and sulfonylureas  (no other diabetes tx) | 53 | 0.5 | <10 |  |
| Diabetes treatment  (no insulin or sulfonylureas) | Diabetes treatment  (no insulin or sulfonylureas) | 422 | 3.8 | 78 | 4.7 |
| Diabetes treatment  (no insulin or sulfonylureas) | Insulin (in comb.  excluding sulfonylureas) | 316 | 2.9 | 80 | 4.9 |
| Diabetes treatment  (no insulin or sulfonylureas) | Sulfonylureas (in comb.  excluding insulin) | 84 | 0.8 | 28 | 1.7 |
| Diabetes treatment  (no insulin or sulfonylureas) | Insulin and sulfonylureas  (comb. with other diabetes tx) | 33 | 0.3 | <10 |  |
| Insulin (in comb.  excluding sulfonylureas) | No medication | 43 | 0.4 | <10 |  |
| Insulin (in comb.  excluding sulfonylureas) | Insulin (alone) | 394 | 3.6 | 38 | 2.3 |
| Insulin (in comb.  excluding sulfonylureas) | Sulfonylureas (alone) | <10 |  | <10 |  |
| Insulin (in comb.  excluding sulfonylureas) | Insulin and sulfonylureas  (no other diabetes tx) | <10 |  | <10 |  |
| Insulin (in comb.  excluding sulfonylureas) | Diabetes treatment  (no insulin or sulfonylureas) | 16 | 0.1 | <10 |  |
| Insulin (in comb.  excluding sulfonylureas) | Insulin (in comb.  excluding sulfonylureas) | 120 | 1.1 | 14 | 0.9 |
| Insulin (in comb.  excluding sulfonylureas) | Sulfonylureas (in comb.  excluding insulin) | <10 |  | <10 |  |
| Insulin (in comb.  excluding sulfonylureas) | Insulin and sulfonylureas  (comb. with other diabetes tx) | <10 |  | <10 |  |
| Sulfonylureas (in comb.  excluding insulin) | No medication | 106 | 1.0 | <10 |  |
| Sulfonylureas (in comb.  excluding insulin) | Insulin (alone) | 685 | 6.2 | 70 | 4.3 |
| Sulfonylureas (in comb.  excluding insulin) | Sulfonylureas (alone) | 40 | 0.4 | <10 |  |
| Sulfonylureas (in comb.  excluding insulin) | Insulin and sulfonylureas  (no other diabetes tx) | 40 | 0.4 | <10 |  |
| Sulfonylureas (in comb.  excluding insulin) | Diabetes treatment  (no insulin or sulfonylureas) | 34 | 0.3 | <10 |  |
| Sulfonylureas (in comb.  excluding insulin) | Insulin (in comb.  excluding sulfonylureas) | 80 | 0.7 | <10 |  |
| Sulfonylureas (in comb.  excluding insulin) | Sulfonylureas (in comb.  excluding insulin) | 84 | 0.8 | 12 | 0.7 |
| Sulfonylureas (in comb.  excluding insulin) | Insulin and sulfonylureas  (comb. with other diabetes tx) | 80 | 0.7 | <10 |  |
| Insulin and sulfonylureas  (comb. with other diabetes tx) | No medication | 17 | 0.2 | <10 |  |
| Insulin and sulfonylureas  (comb. with other diabetes tx) | Insulin (alone) | 137 | 1.2 | 12 | 0.7 |
| Insulin and sulfonylureas  (comb. with other diabetes tx) | Sulfonylureas (alone) | <10 |  | <10 |  |
| Insulin and sulfonylureas  (comb. with other diabetes tx) | Insulin and sulfonylureas  (no other diabetes tx) | <10 |  | <10 |  |
| Insulin and sulfonylureas  (comb. with other diabetes tx) | Diabetes treatment  (no insulin or sulfonylureas) | <10 |  | <10 |  |
| Insulin and sulfonylureas  (comb. with other diabetes tx) | Insulin (in comb.  excluding sulfonylureas) | 28 | 0.3 | <10 |  |
| Insulin and sulfonylureas  (comb. with other diabetes tx) | Sulfonylureas (in comb.  excluding insulin) | <10 |  | <10 |  |
| Insulin and sulfonylureas  (comb. with other diabetes tx) | Insulin and sulfonylureas  (comb. with other diabetes tx) | 21 | 0.2 | <10 |  |

*Cell counts of 10 or less are suppressed to protect patient privacy

Supplemental figures augmenting the main analysis (S1-S4)


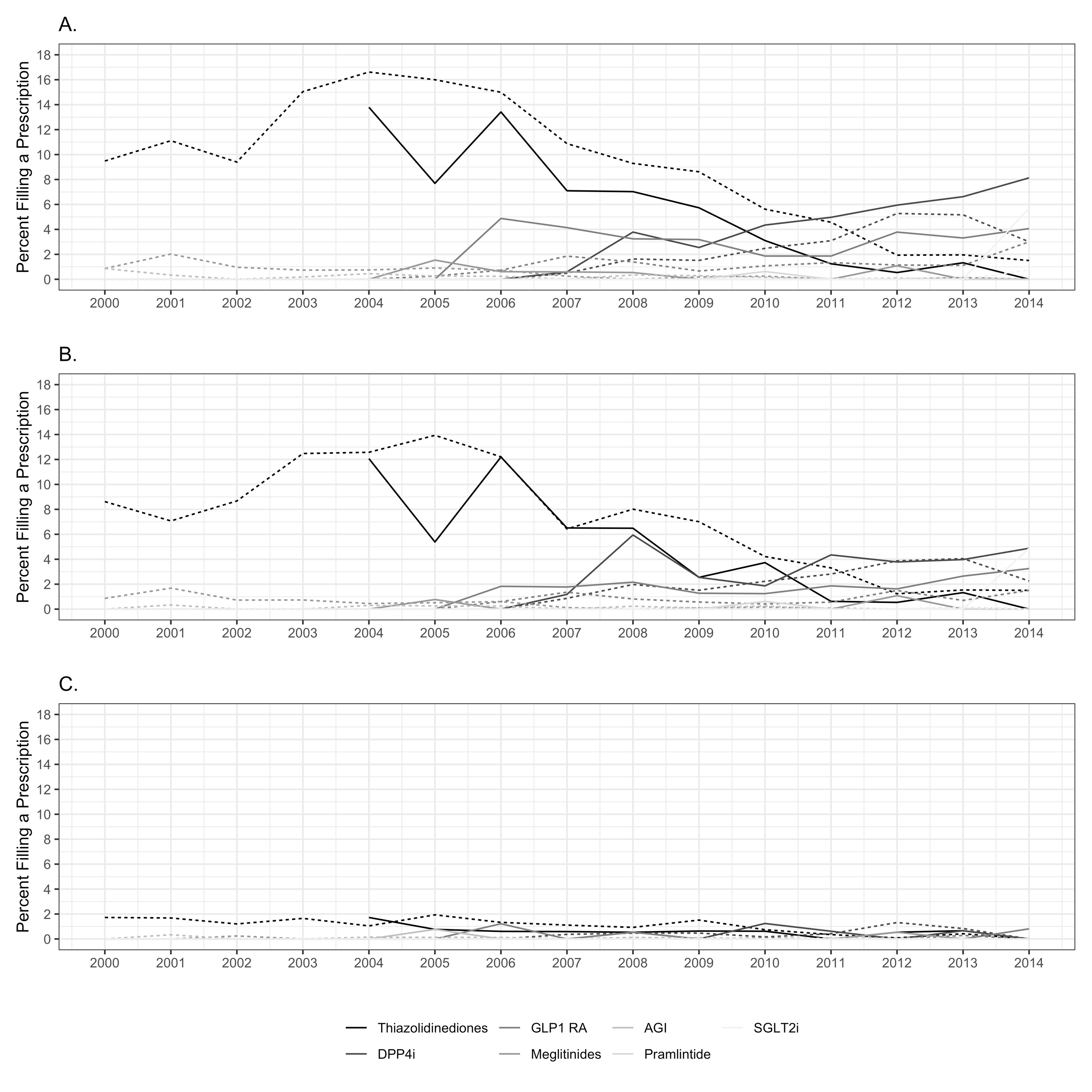


**Supplemental Figure S1.** Secular trends in use of specific other non-insulin antidiabetic medications by women with type 2 diabetes (A) before pregnancy (B) during the first trimester and (C) during the second trimester. Dotted lines show proportions among 10,987 Medicaid women (2000 – 2014) and solid lines show proportions among 1,644 privately insured women (2004 – 2014). Medications may be used alone or in combination.

**
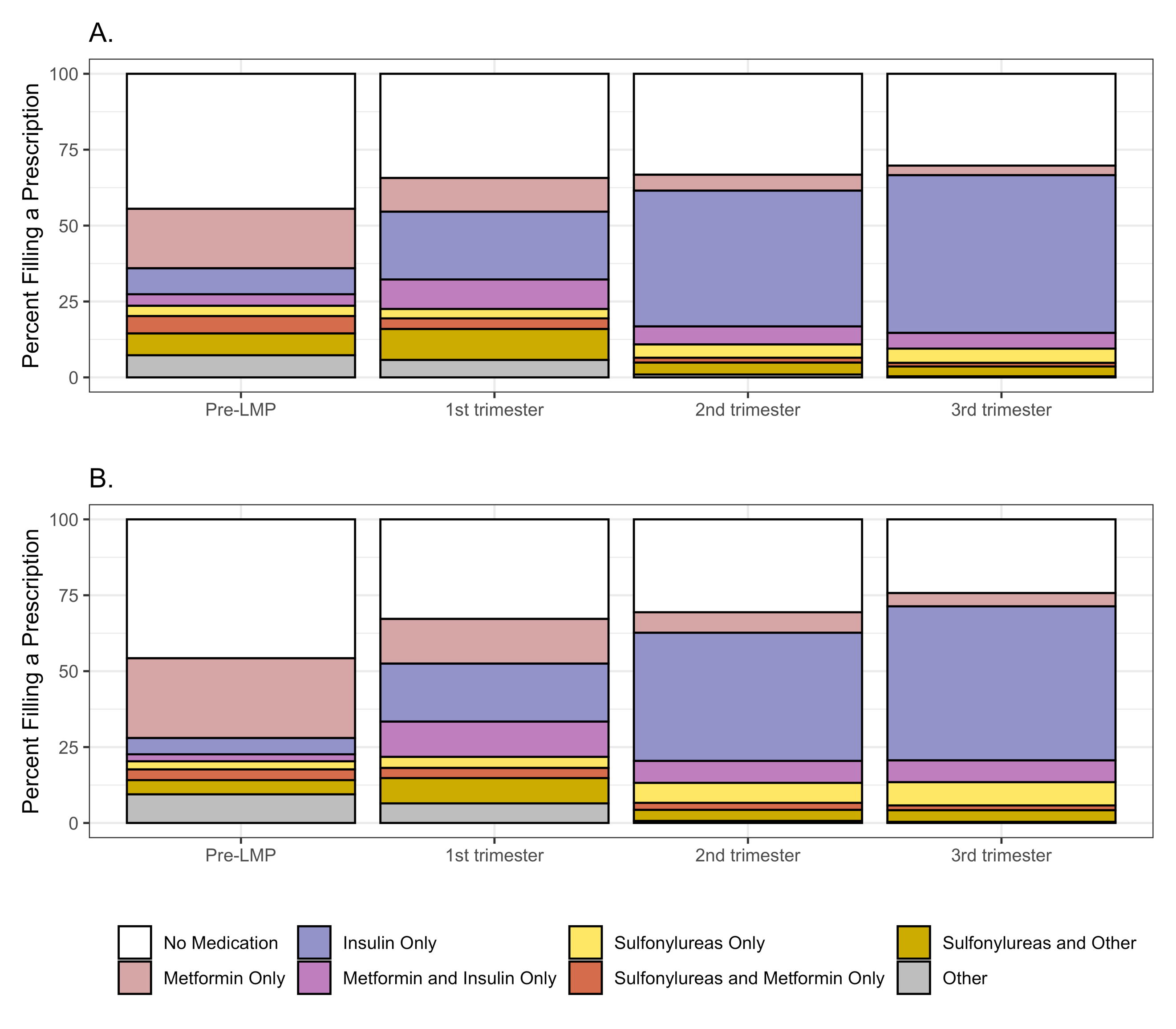
**

**Supplemental Figure S2.** Proportion of users in mutually exclusive treatment categories, before pregnancy (pre-LMP) and in each trimester, (A) among 10,987 Medicaid women (2000 – 2014) and (B) among 1,644 privately insured women (2004 – 2014). “Other” includes thiazolidinediones, AGI, SGLT2i, DPP4i, GLP1 RA, and meglitinides.


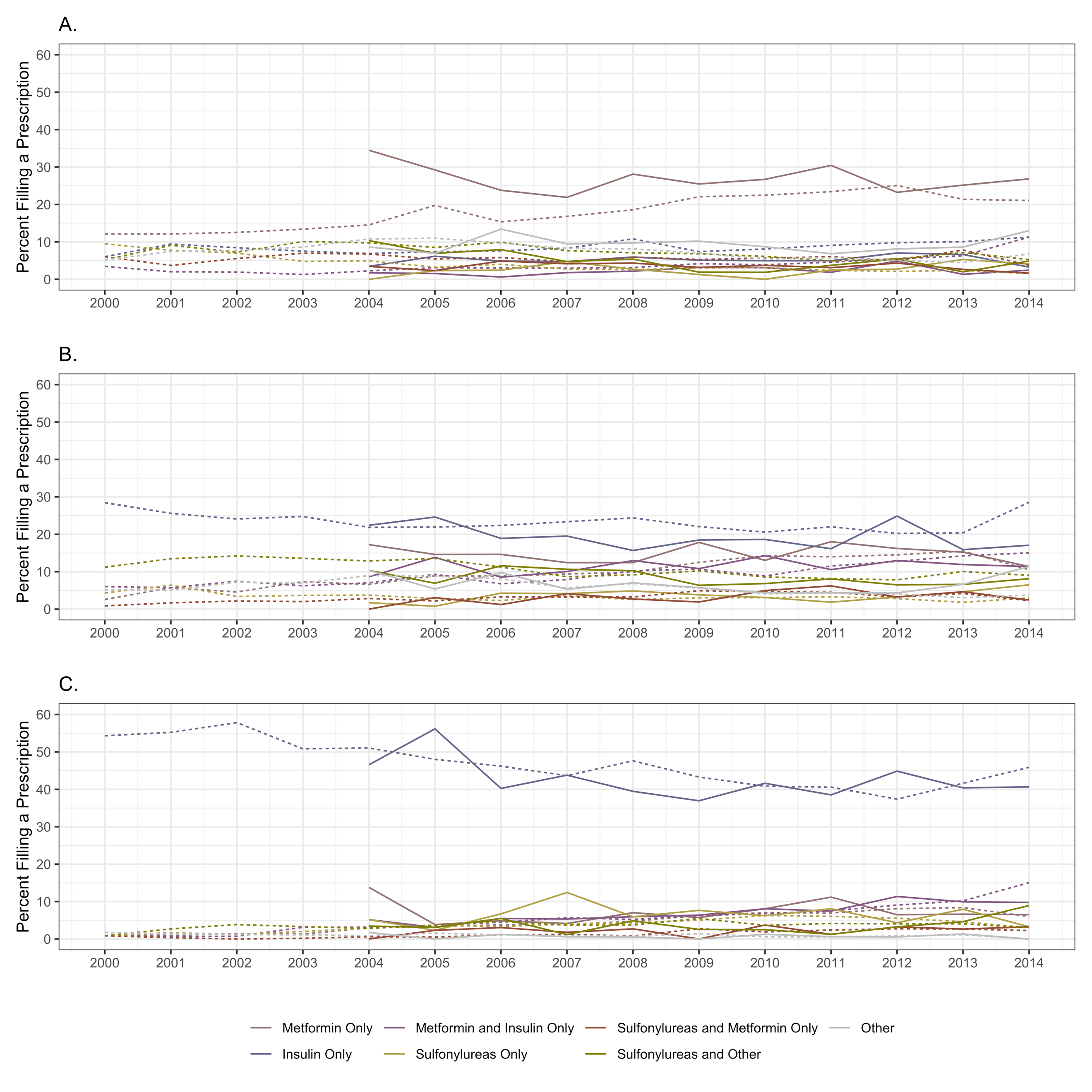


**Supplemental Figure S3.** Secular trends in treatment strategies for type 2 diabetes (A) before pregnancy (B) during the first trimester and (C) during the second trimester. Dotted lines show proportions in mutually exclusive treatment categories among 10,987 Medicaid women (2000 – 2014) and solid lines show proportions among 1,644 privately insured women (2004 – 2014). Medications may be used alone or in combination. “Other” includes thiazolidinediones, AGI, SGLT2i, DPP4i, GLP1 RA, pramlintide, and meglitinides. “No treatment” group has been excluded.


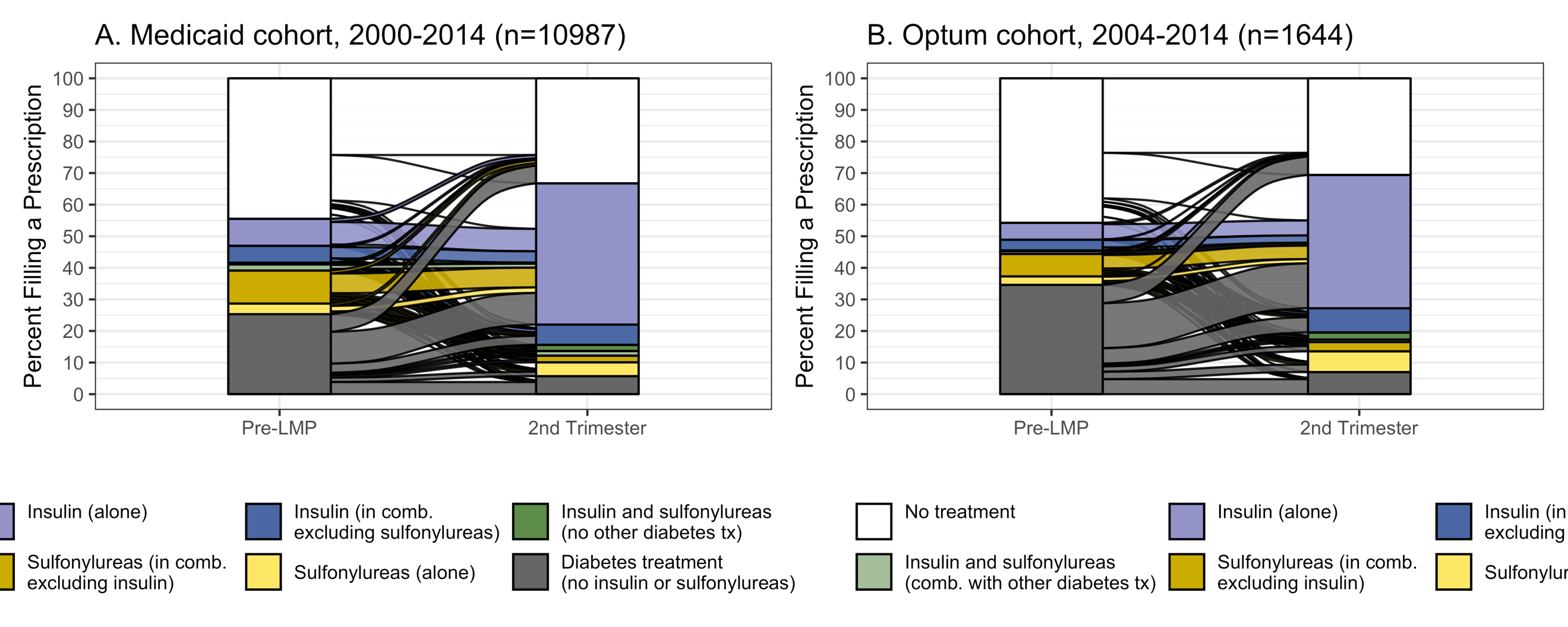


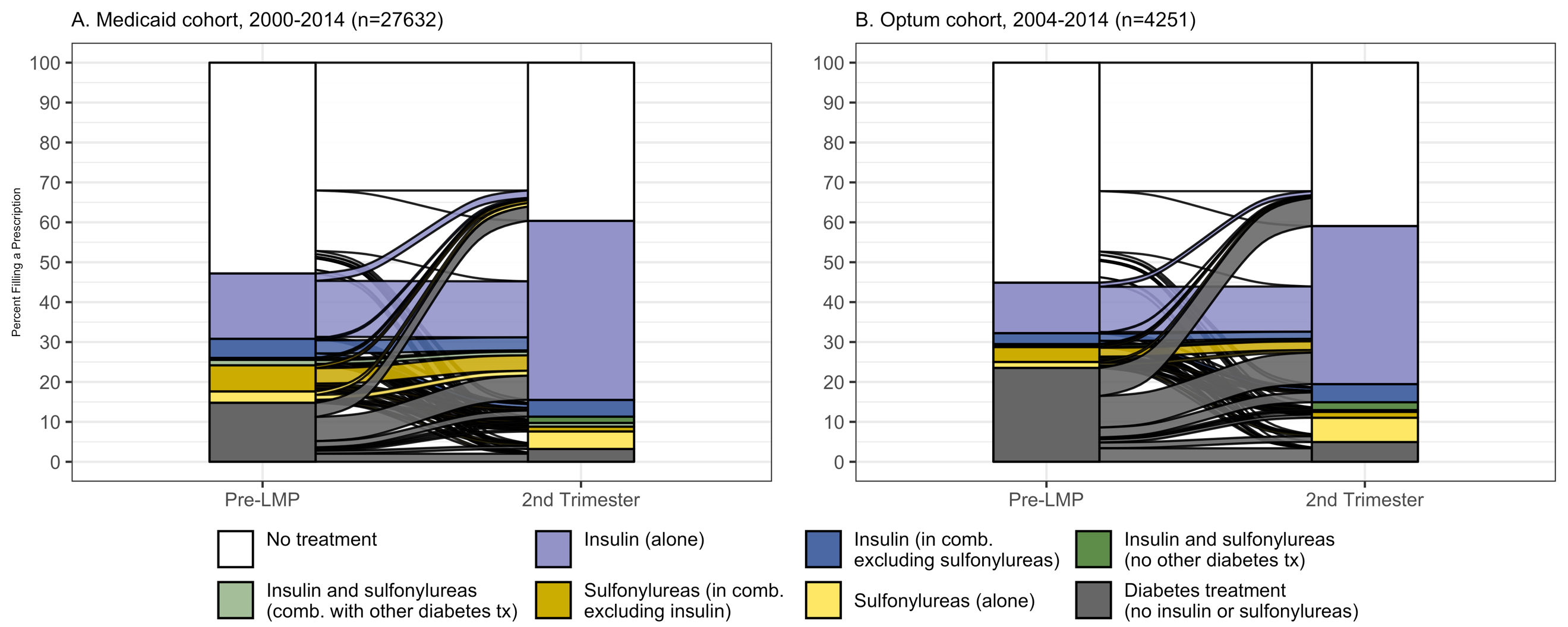


**Supplemental Figure S4.** Longitudinal patterns in insulin and sulfonylurea treatment from before pregnancy to the second trimester. Horizontal flows show the proportion of users of a treatment strategy, alone or in combination, as use changes. “Diabetes treatment” references any diabetes medications, alone or in combination, not including insulin or sulfonylurea. Panel A shows proportions among 10,987 Medicaid women (2000 – 2014) and panel B shows proportions among 1,644 privately insured women (2004 – 2014).

Sensitivity analyses examining longitudinal patterns before and after 2008 (S5-S10)


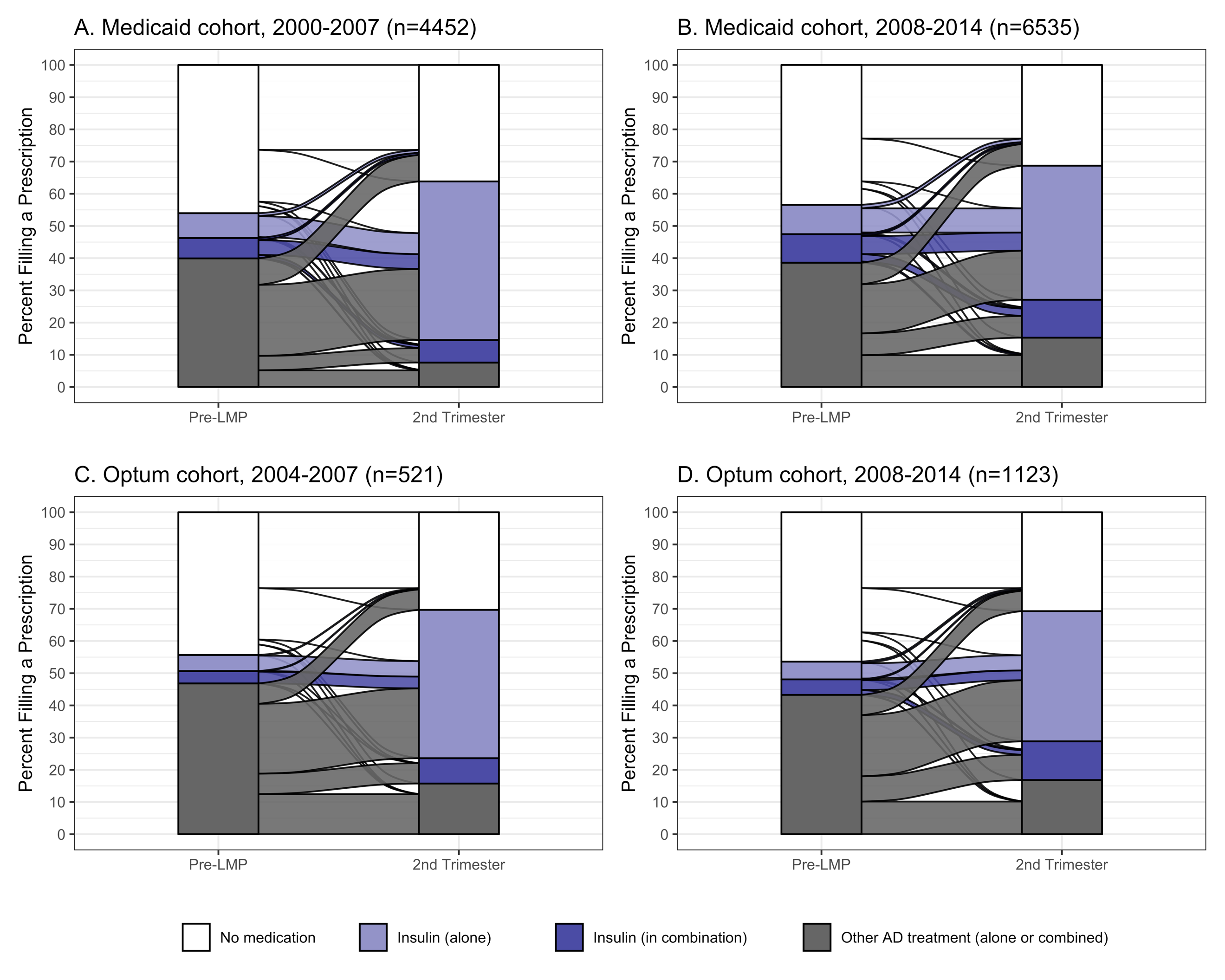


**Supplemental Figure S5.** Longitudinal patterns in insulin treatment from before pregnancy to the second trimester. Horizontal flows show the proportion of users of a treatment strategy, alone or in combination, as use changes. “Other AD treatment” references any diabetes medications, alone or in combination, not including insulin. Panels A and B show proportions in Medicaid from 2000 – 2007 and 2008 – 2014, and panels C and D show proportions among privately insured women from 2004 – 2007 and 2008 – 2014.


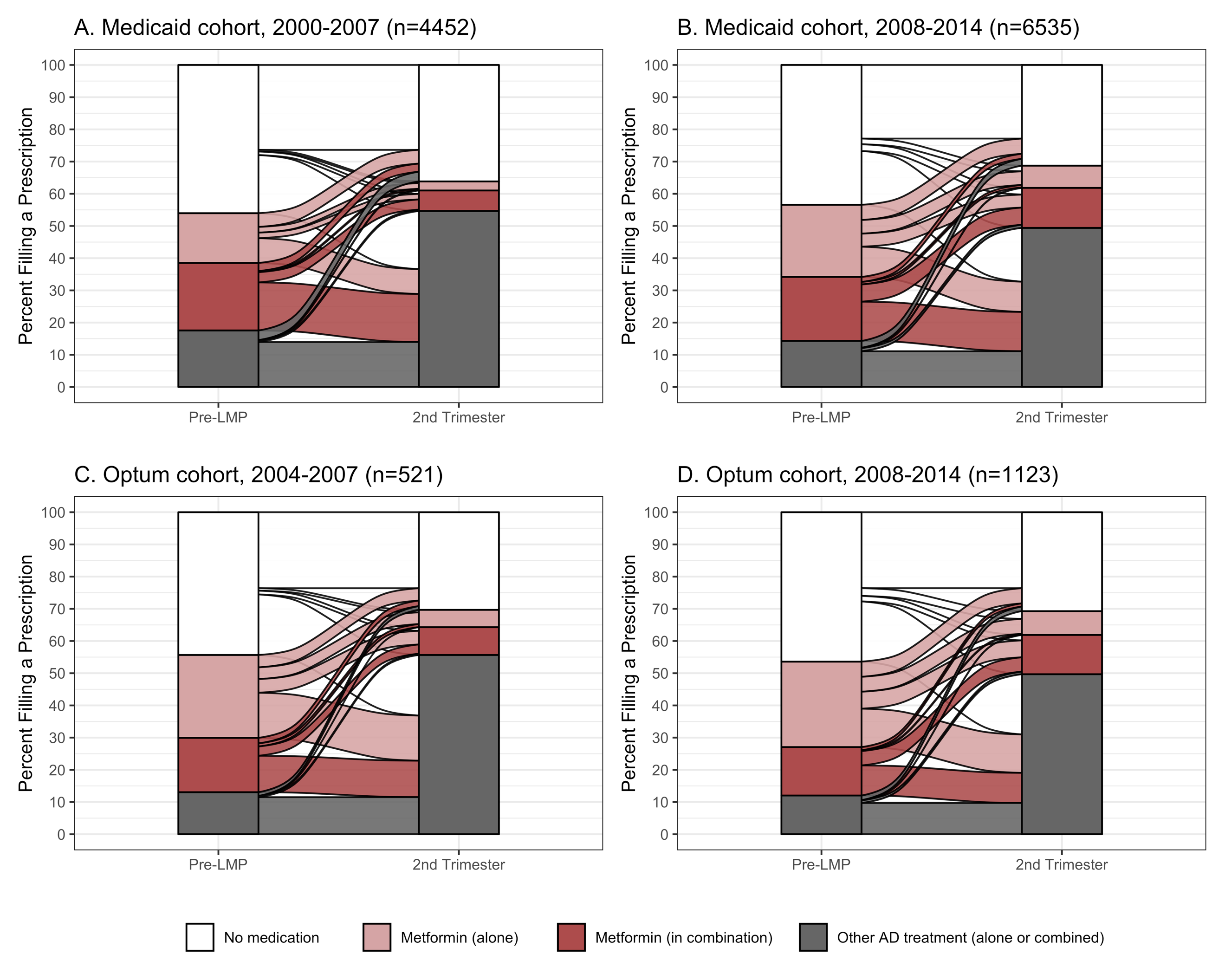


**Supplemental Figure S6.** Longitudinal patterns in metformin treatment from before pregnancy to the second trimester. Horizontal flows show the proportion of users of a treatment strategy, alone or in combination, as use changes. “Other AD treatment” references any diabetes medications, alone or in combination, not including metformin. Panels A and B show proportions in Medicaid from 2000 – 2007 and 2008 – 2014, and panels C and D show proportions among privately insured women from 2004 – 2007 and 2008 – 2014.


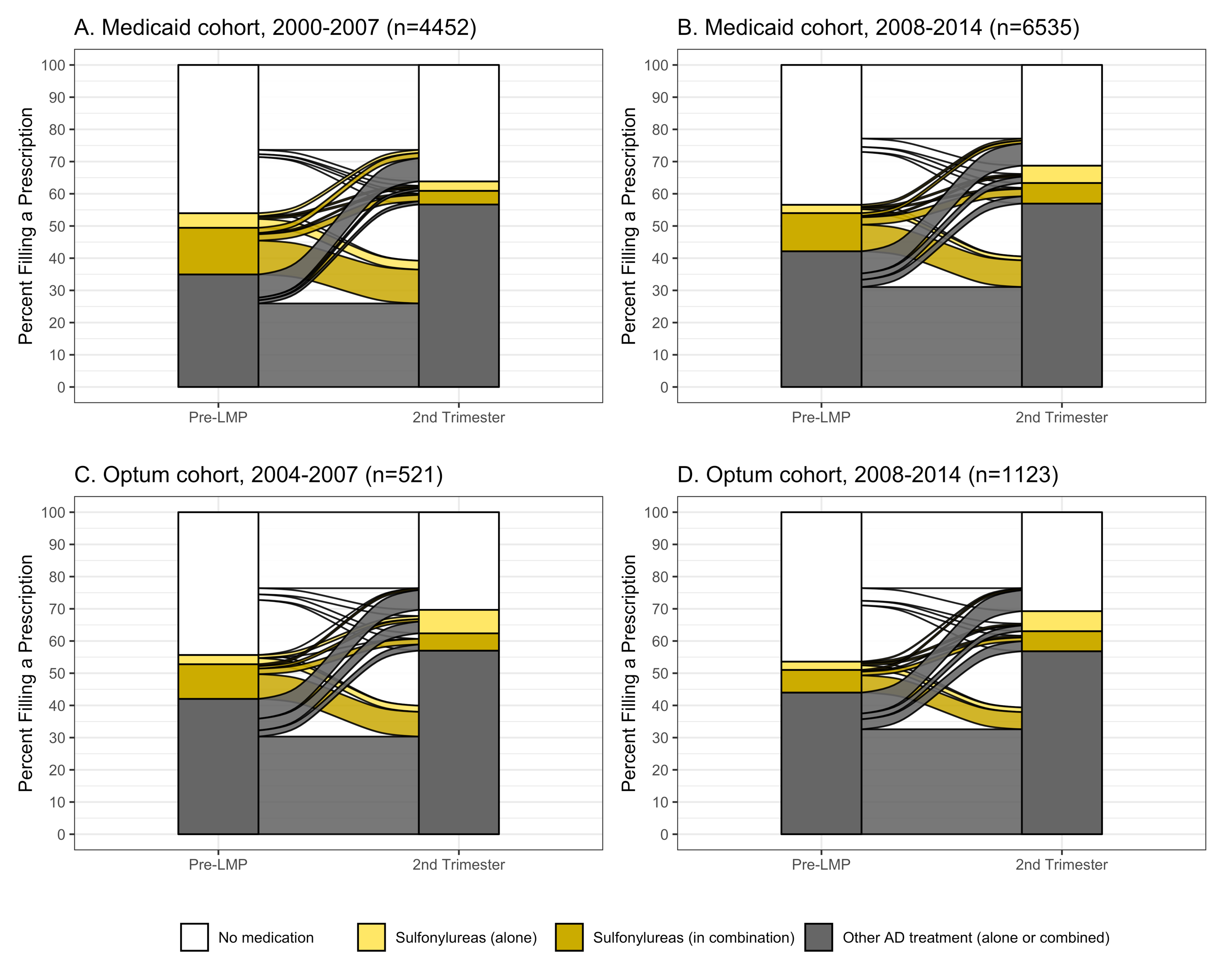


**Supplemental Figure S7.** Longitudinal patterns in sulfonylurea treatment from before pregnancy to the second trimester. Horizontal flows show the proportion of users of a treatment strategy, alone or in combination, as use changes. “Other AD treatment” references any diabetes medications, alone or in combination, not including sulfonylureas. Panels A and B show proportions in Medicaid from 2000 – 2007 and 2008 – 2014, and panels C and D show proportions among privately insured women from 2004 – 2007 and 2008 – 2014.


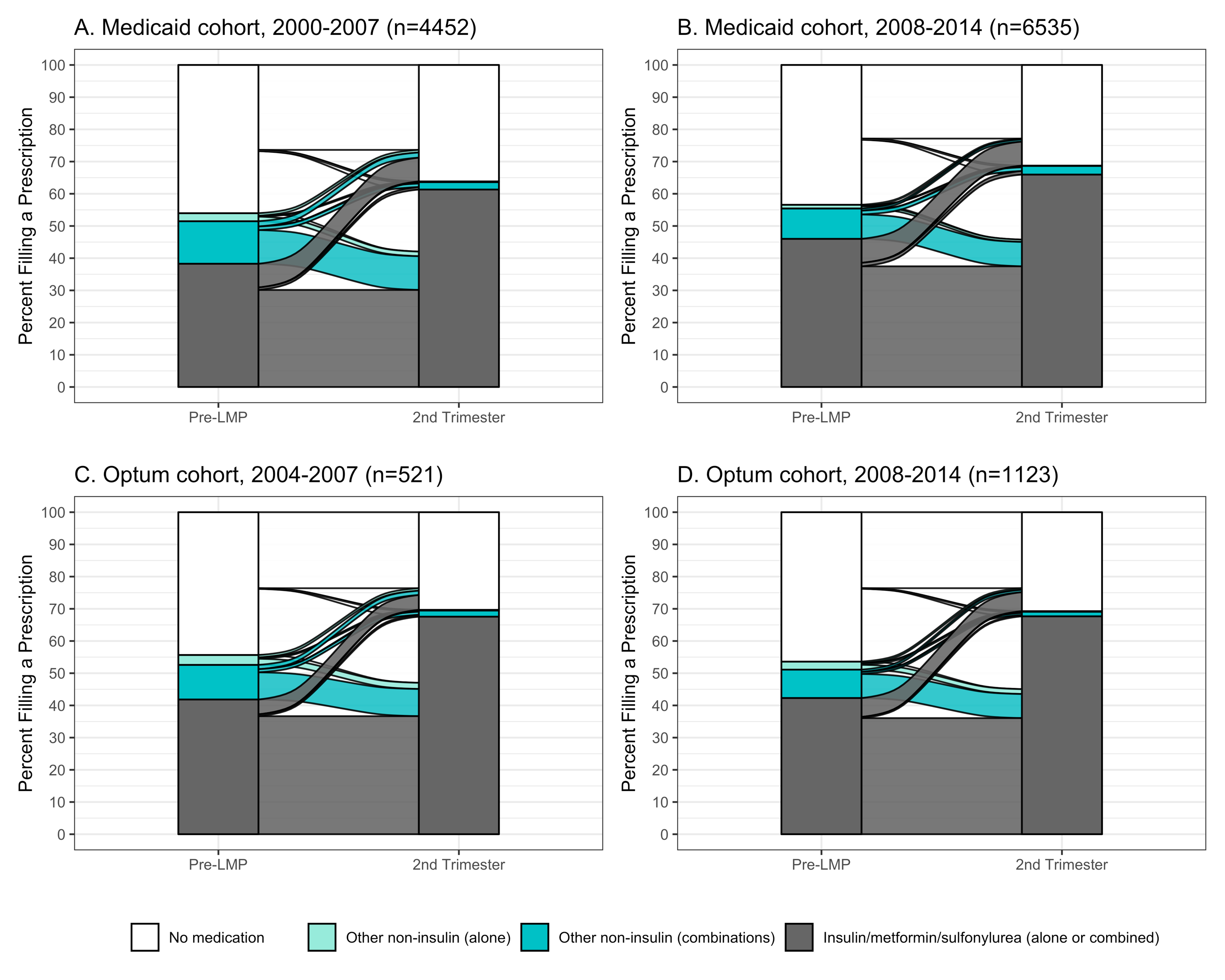


**Supplemental Figure S8.** Longitudinal patterns in other non-insulin antidiabetic treatment, including thiazolidinediones, AGI, SGLT2i, DPP4i, GLP1 RA, pramlintide, and meglitinides, from before pregnancy to the second trimester. Horizontal flows show the proportion of users of a treatment strategy, alone or in combination, as use changes. “Insulin/metformin/sulfonylurea” references any diabetes medications, alone or in combination, not including other non-insulin antidiabetics. Panels A and B show proportions in Medicaid from 2000 – 2007 and 2008 – 2014, and panels C and D show proportions among privately insured women from 2004 – 2007 and 2008 – 2014.


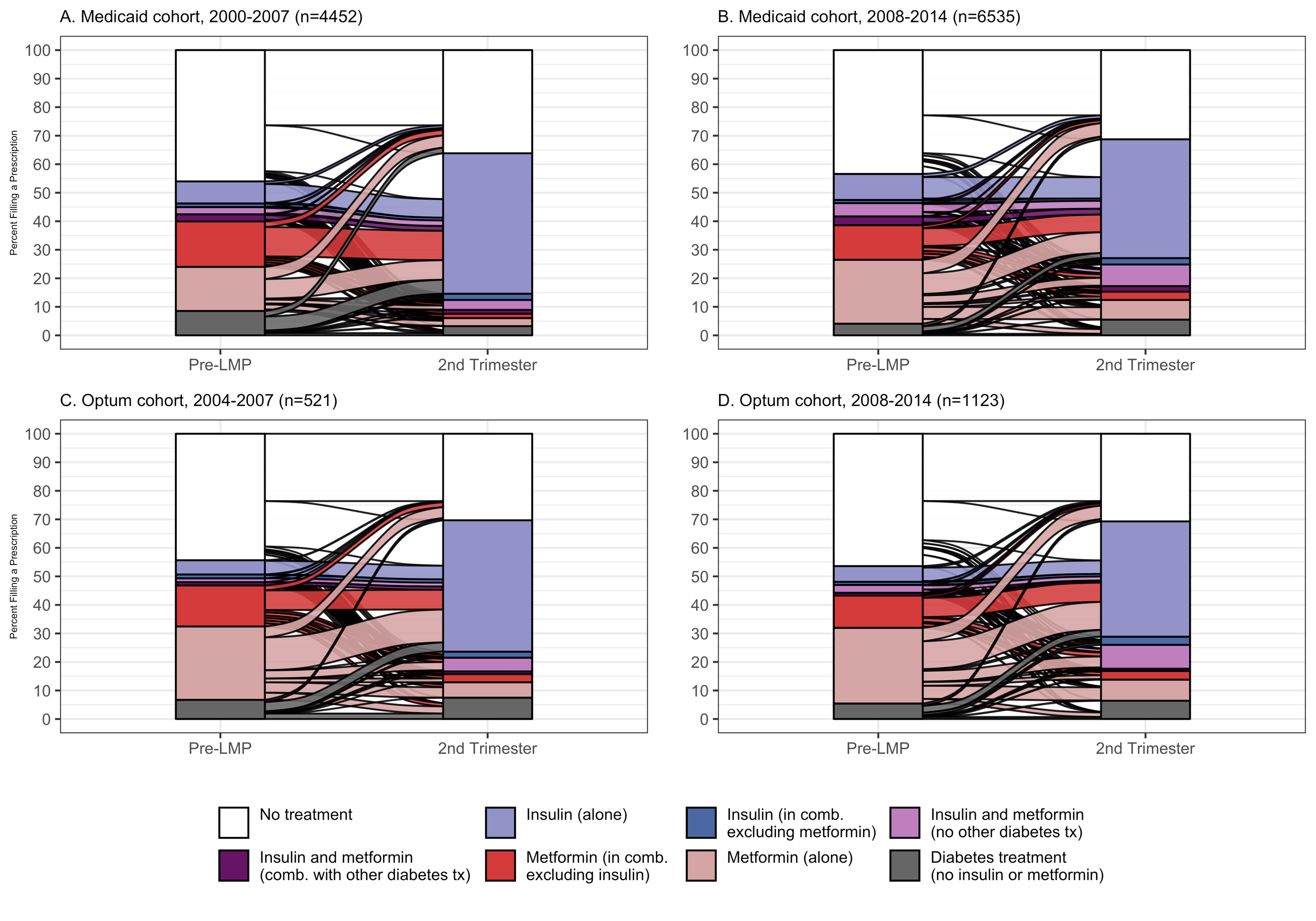


**Supplemental Figure S9.** Longitudinal patterns in insulin and sulfonylurea treatment from before pregnancy to the second trimester. Horizontal flows show the proportion of users of a treatment strategy, alone or in combination, as use changes. “Diabetes treatment” references any diabetes medications, alone or in combination, not including insulin or sulfonylurea. Panels A and B show proportions in Medicaid from 2000 – 2007 and 2008 – 2014, and panels C and D show proportions among privately insured women from 2004 – 2007 and 2008 – 2014.

**
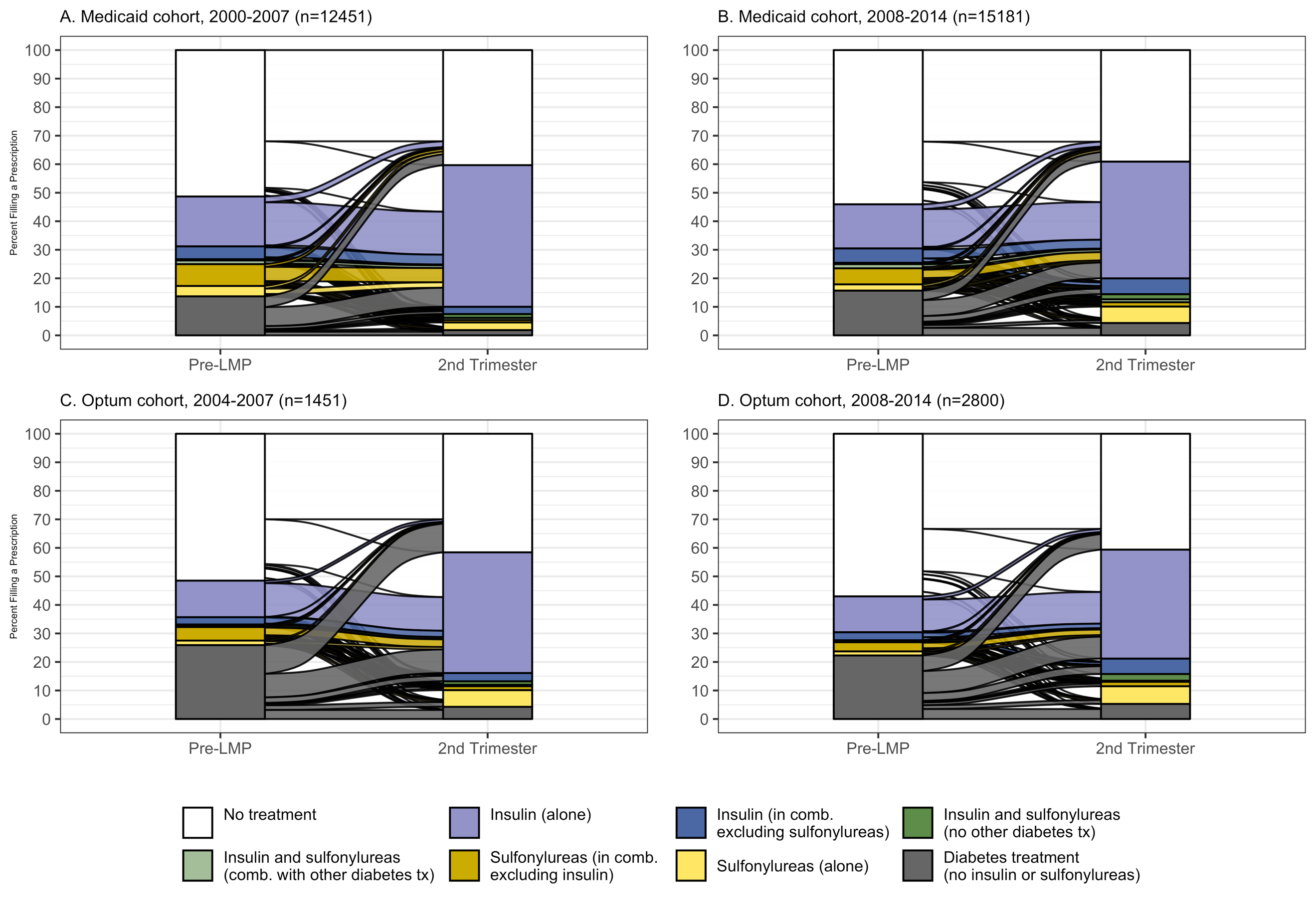
**

**Supplemental Figure S10.** Longitudinal patterns in insulin and sulfonylurea treatment from before pregnancy to the second trimester. Horizontal flows show the proportion of users of a treatment strategy, alone or in combination, as use changes. “Diabetes treatment” references any diabetes medications, alone or in combination, not including insulin or sulfonylurea. Panels A and B show proportions in Medicaid from 2000 – 2007 and 2008 – 2014, and panels C and D show proportions among privately insured women from 2004 – 2007 and 2008 – 2014.

Sensitivity analysis showing results from alternate type 2 definition (S11-S18)


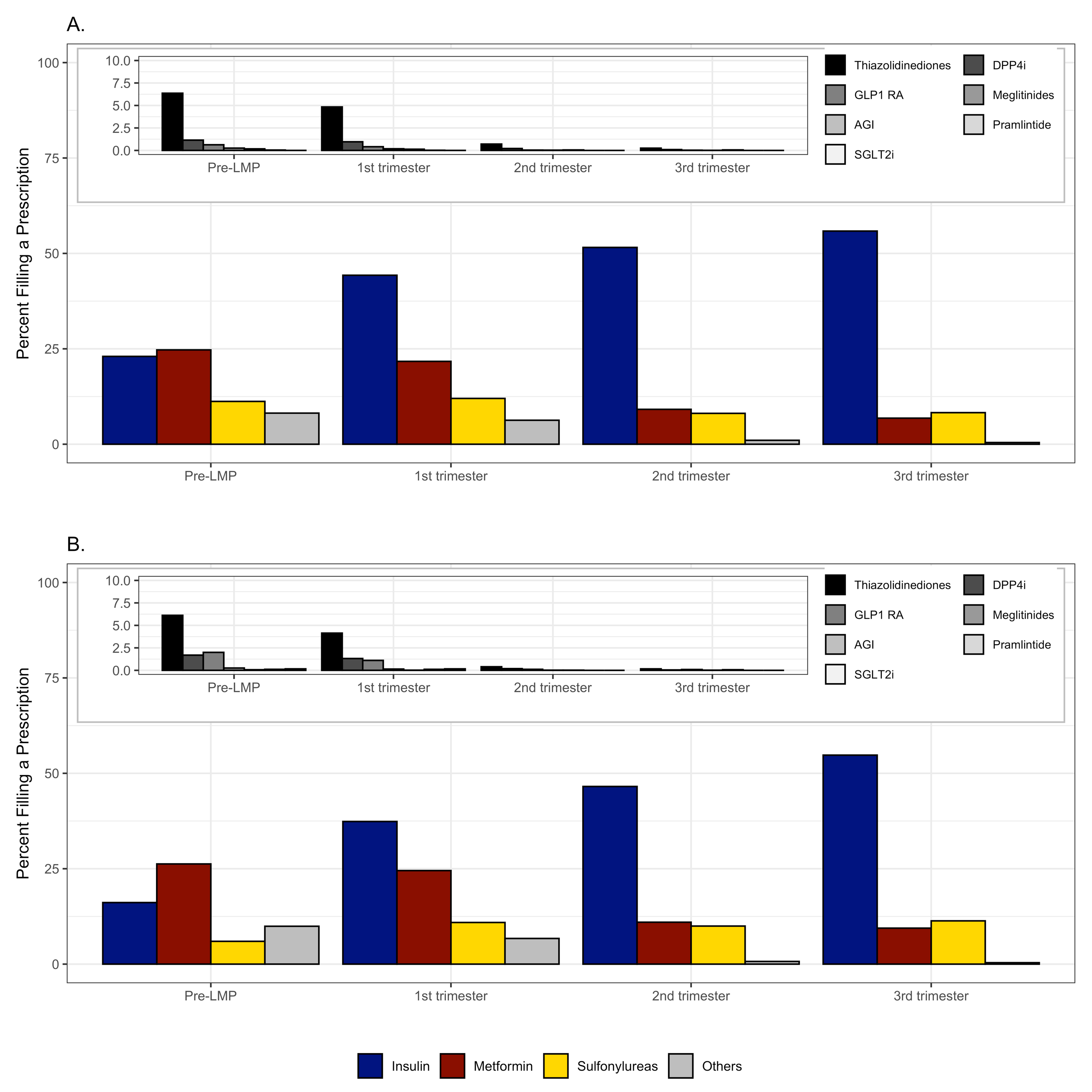


**Supplemental Figure S11**. Proportion of women with type 2 diabetes using antidiabetic medications before and during pregnancy (A) among 27,632 Medicaid women (2000 – 2014) and (B) among 4,251 privately insured women (2004 – 2014). Inset plots show proportion of users for medications classified as “others” in the main plot. Pre-LMP refers to the 180 days before the last menstrual period.


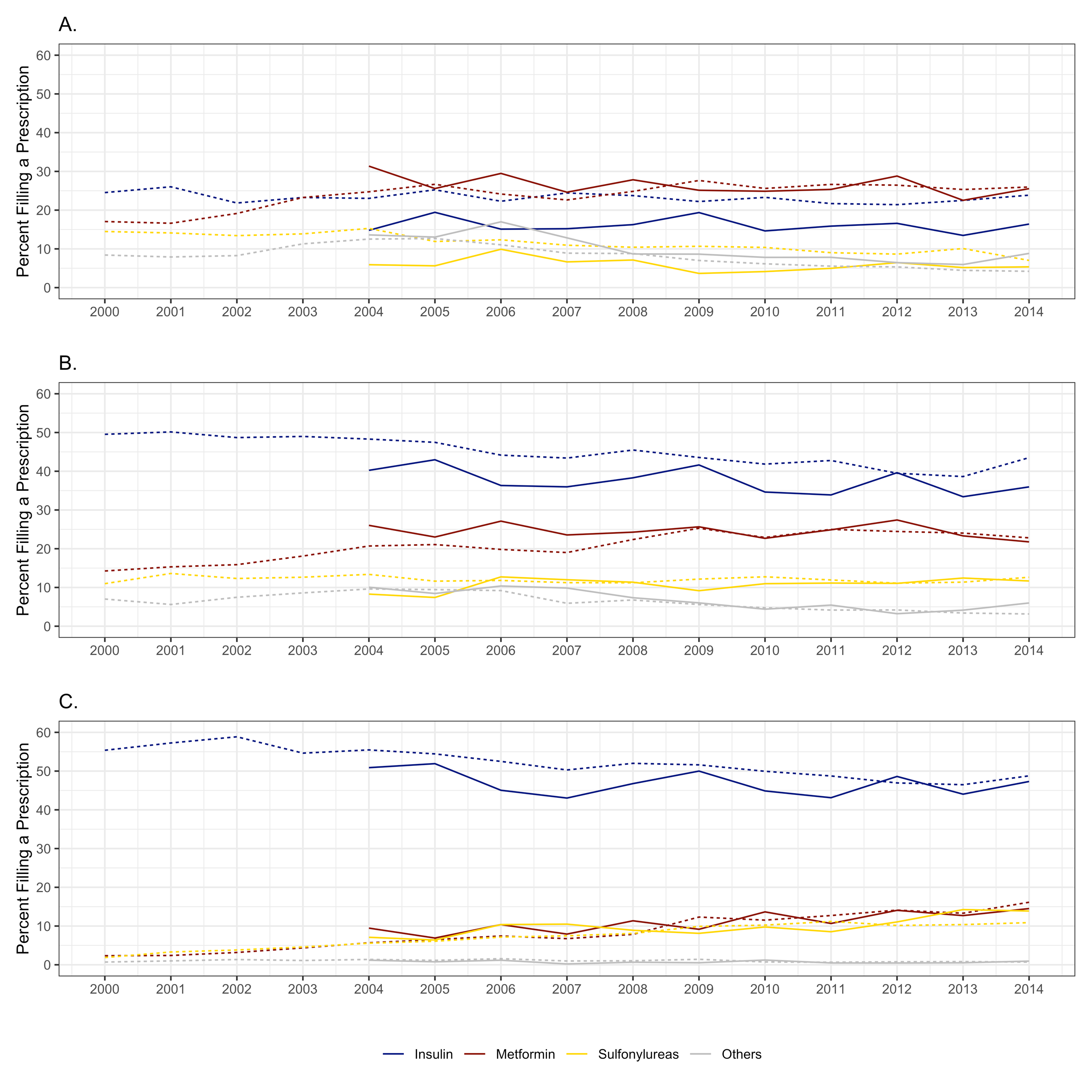


**Supplemental Figure S12.** Secular trends in use of antidiabetic medications by women with type 2 diabetes (A) before pregnancy (B) during the first trimester and (C) during the second trimester. Dotted lines show proportions among 27,632 Medicaid women (2000 – 2014) and solid lines show proportions among 4,251 privately insured women (2004 – 2014). Medications may be used alone or in combination. “Others” includes thiazolidinediones, AGI, SGLT2i, DPP4i, GLP1 RA, pramlintide, and meglitinides.


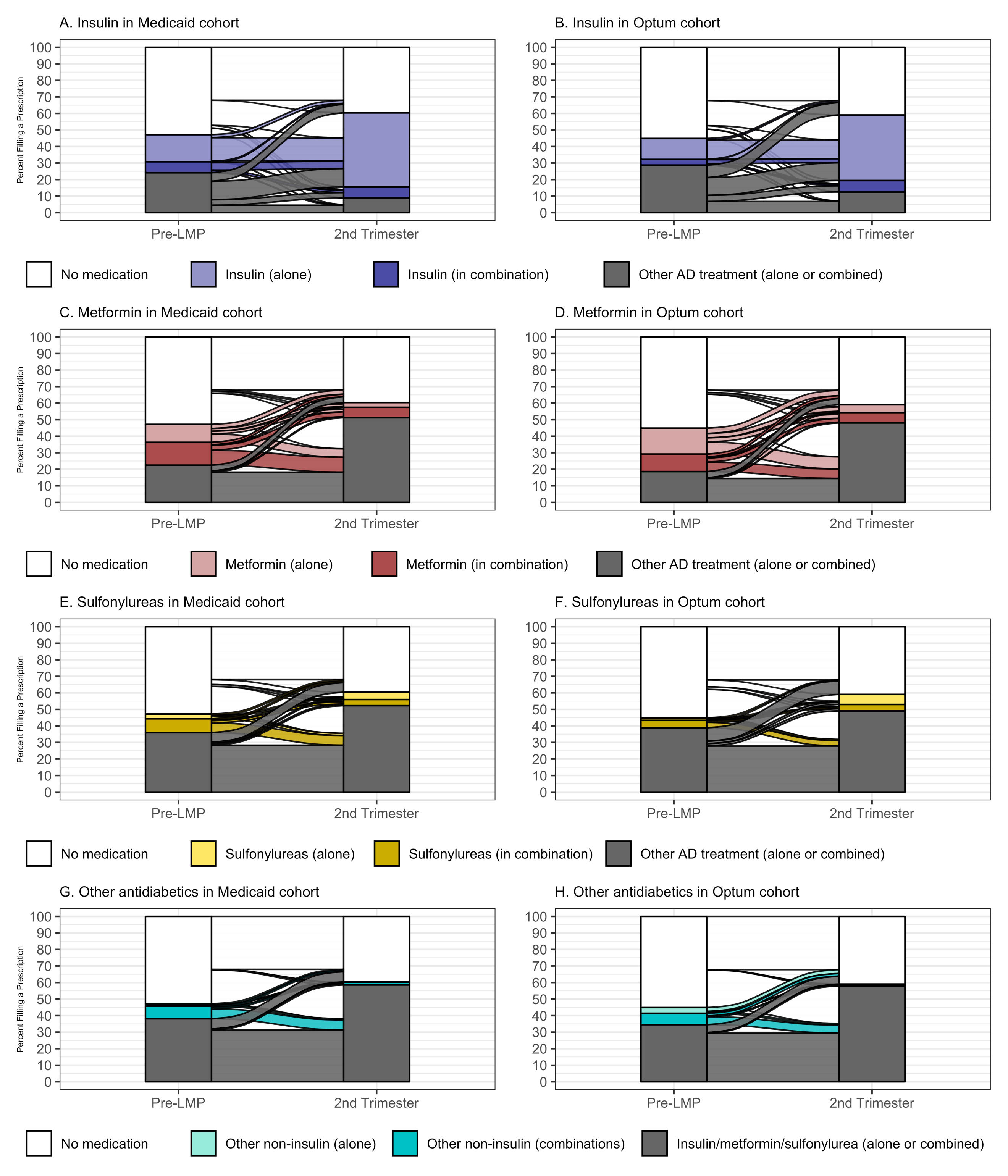


**Supplemental Figure S13.** Longitudinal patterns in antidiabetic medications from before pregnancy to the second trimester. Horizontal flows show the proportion of users of a given drug, alone or in combination, as use changes. “Other AD treatment” is specific to each drug or class and references any diabetes medication not including that drug. “Other non-insulin” (panels G and H) which includes thiazolidinediones, AGI, SGLT2i, DPP4i, GLP1 RA, and meglitinides. Panels A, C, E, and G show proportions among 27,632 Medicaid women (2000 – 2014) and panels B, D, F, and H show proportions among 4,251 privately insured women (2004 – 2014).


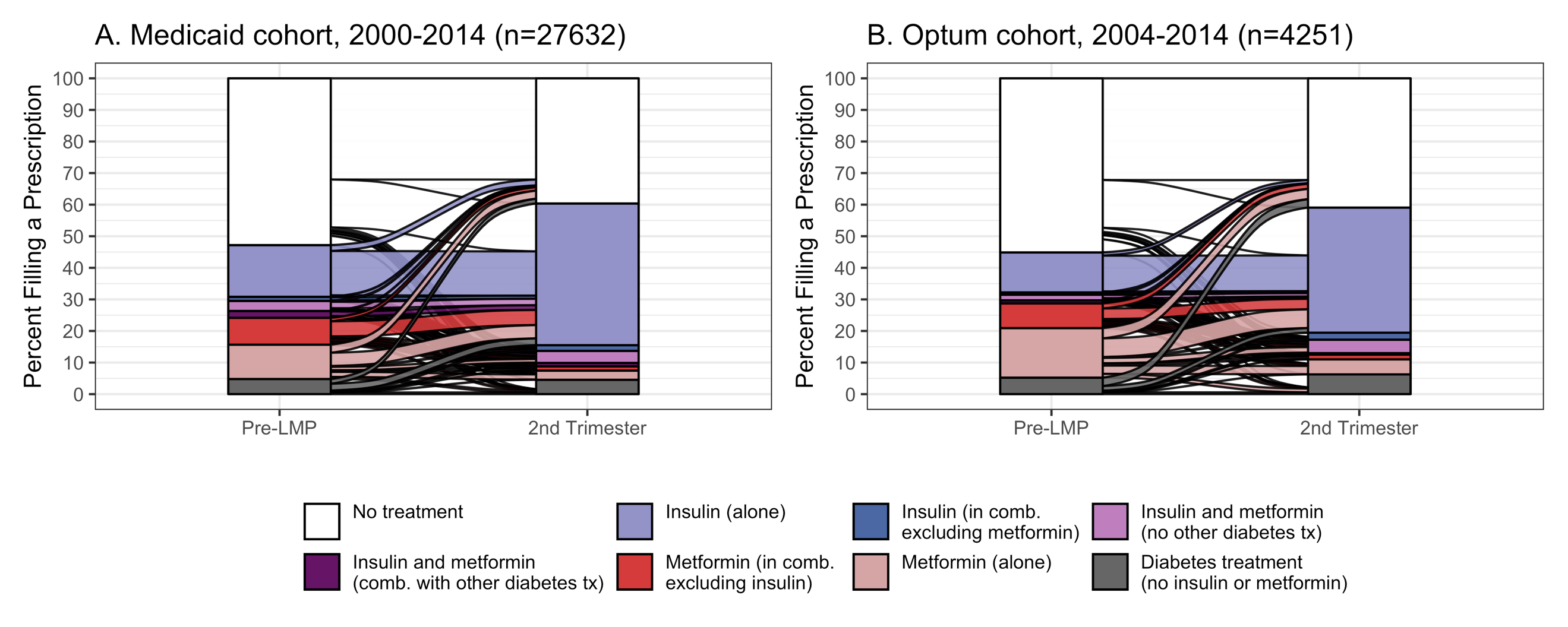


**Supplemental Figure S14.** Longitudinal patterns in insulin and metformin treatment from before pregnancy to the second trimester. Horizontal flows show the proportion of users of a treatment strategy, alone or in combination, as use changes. “Diabetes treatment” references any diabetes medications, alone or in combination, not including insulin or metformin. Panel A shows proportions among 27,632 Medicaid women (2000 – 2014) and panel B shows proportions among 4,251 privately insured women (2004 – 2014).


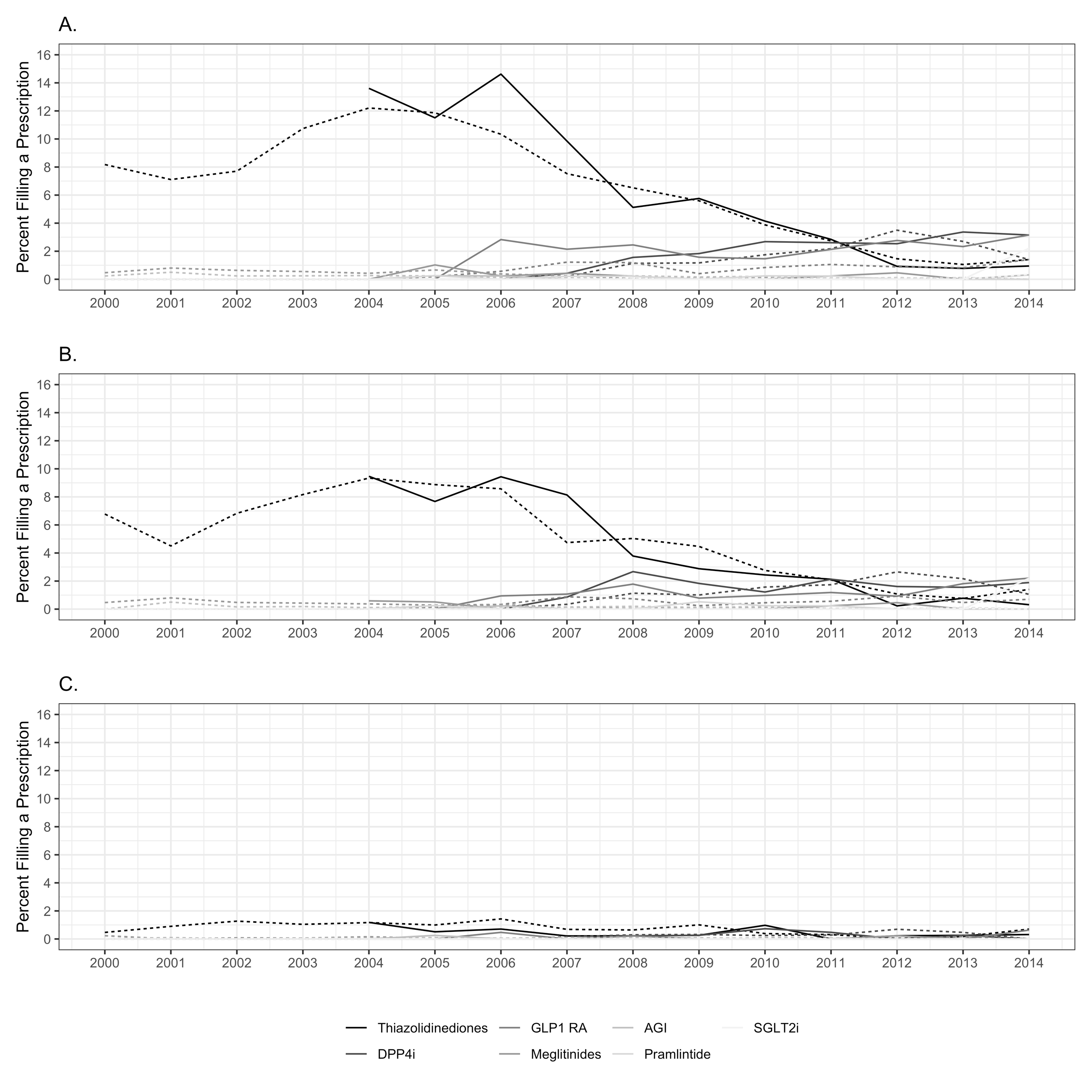


**Supplemental Figure S15.** Secular trends in use of specific other non-insulin antidiabetic medications by women with type 2 diabetes (A) before pregnancy (B) during the first trimester and (C) during the second trimester. Dotted lines show proportions among 27,632 Medicaid women (2000 – 2014) and solid lines show proportions among 4,251 privately insured women (2004 – 2014). Medications may be used alone or in combination.

**
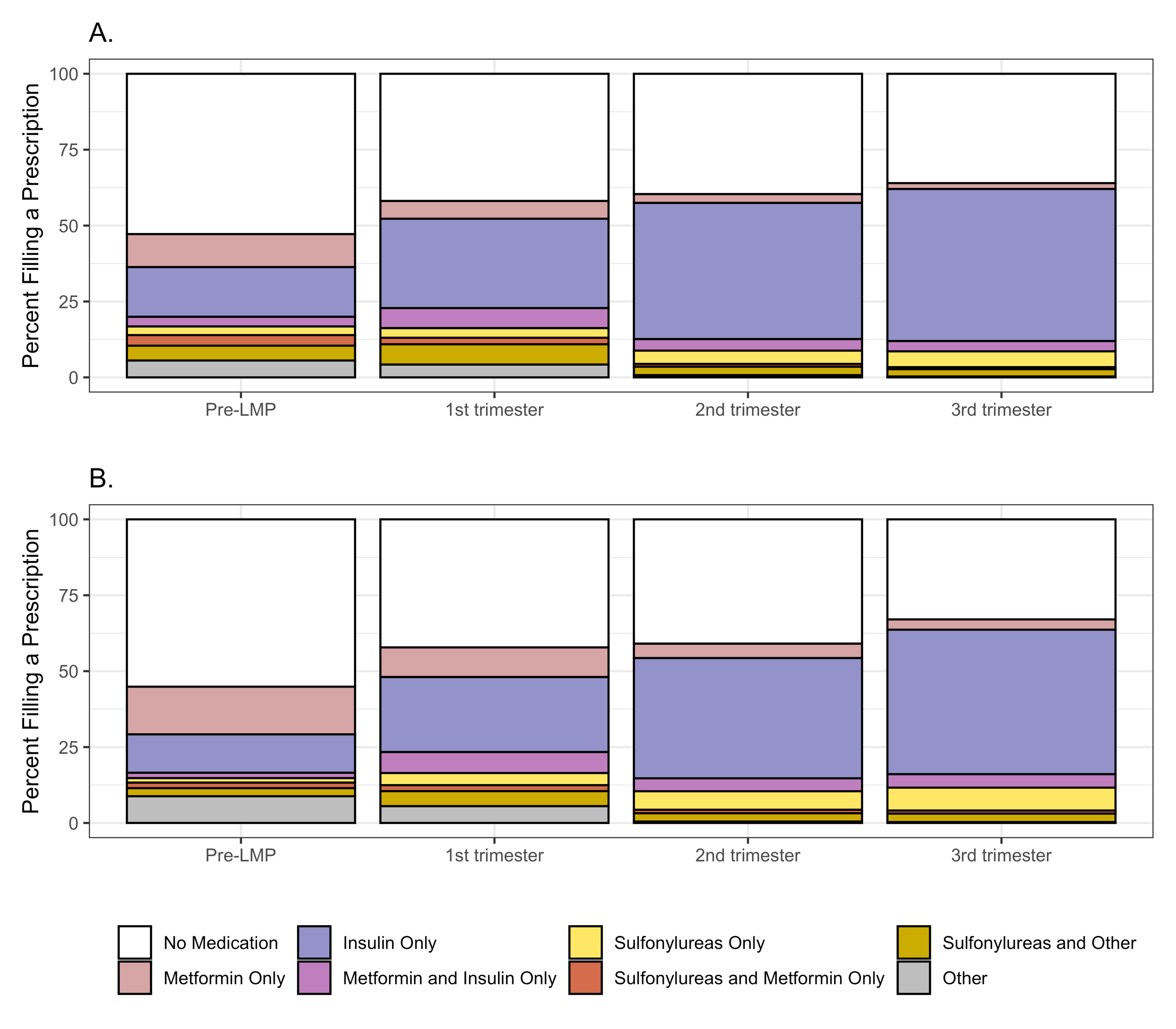
**

**Supplemental Figure S16.** Proportion of users in mutually exclusive treatment categories, before pregnancy (pre-LMP) and in each trimester, (A) among 27,632 Medicaid women (2000 – 2014) and (B) among 4,251 privately insured women (2004 – 2014). “Other” includes thiazolidinediones, AGI, SGLT2i, DPP4i, GLP1 RA, and meglitinides.


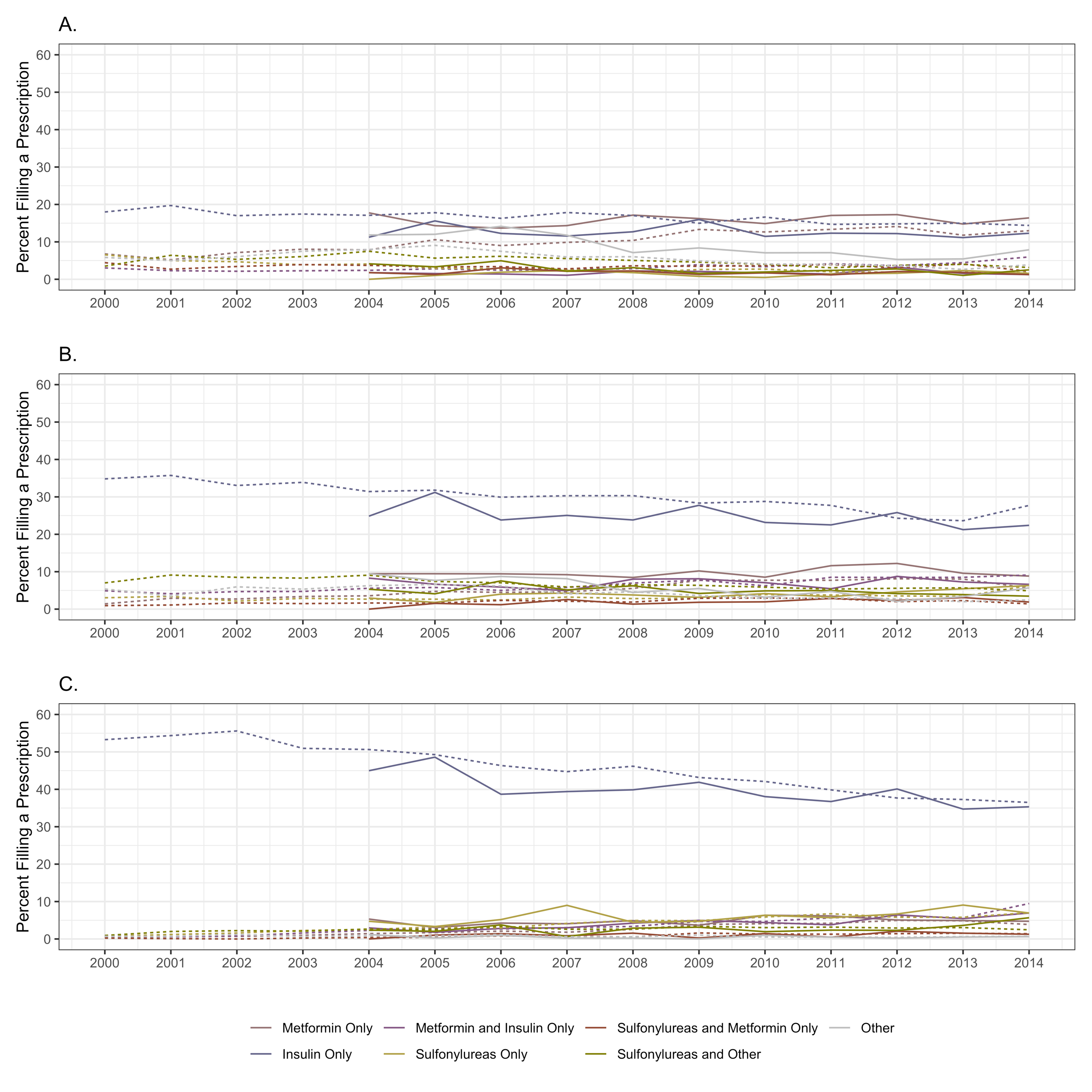


**Supplemental Figure S17.** Secular trends in treatment strategies for type 2 diabetes (A) before pregnancy (B) during the first trimester and (C) during the second trimester. Dotted lines show proportions in mutually exclusive treatment categories among 27,632 Medicaid women (2000 – 2014) and solid lines show proportions among 4,251 privately insured women (2004 – 2014). Medications may be used alone or in combination. “Other” includes thiazolidinediones, AGI, SGLT2i, DPP4i, GLP1 RA, pramlintide, and meglitinides. “No treatment” group has been excluded.

**
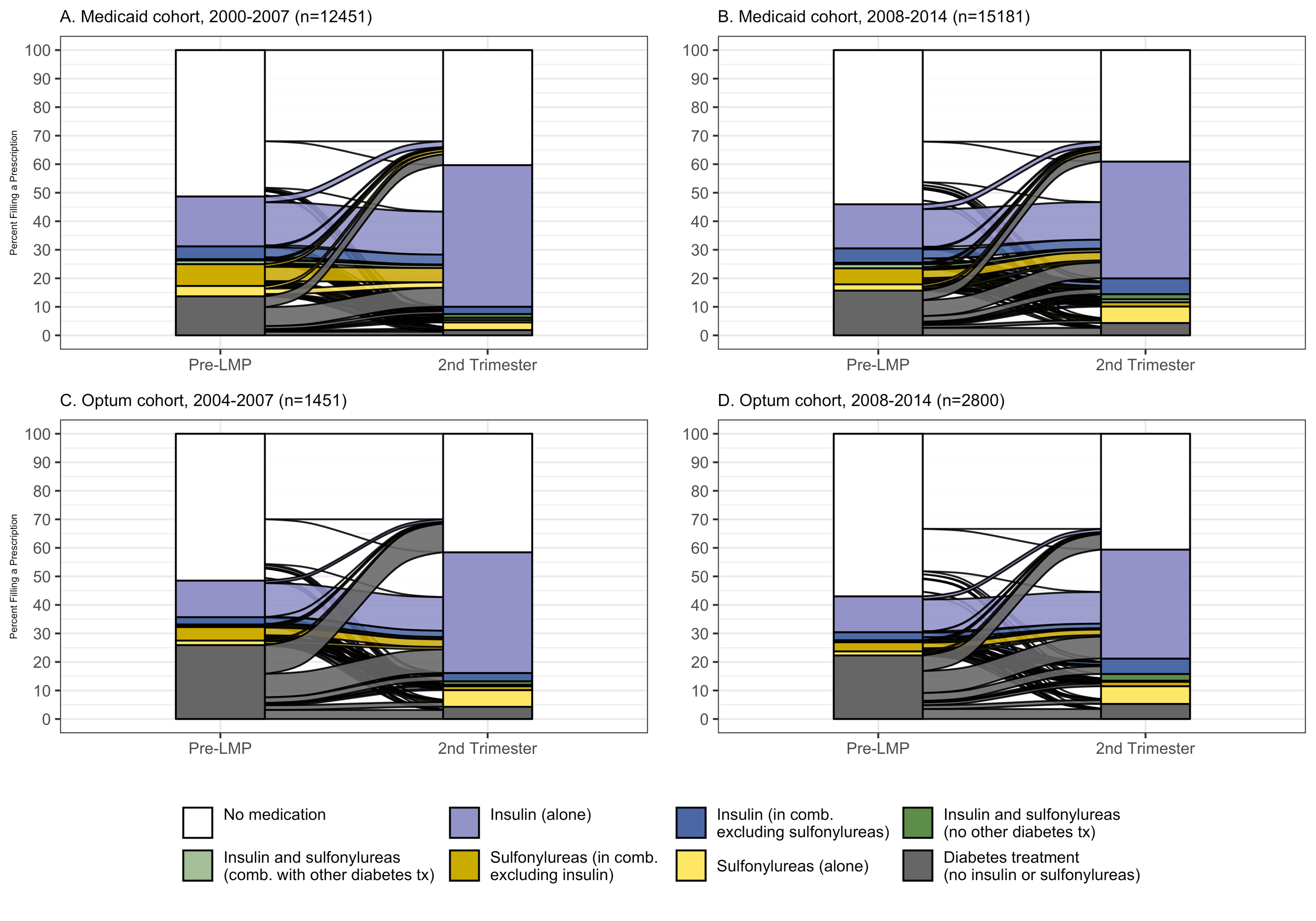
**

**Supplemental Figure S18.** Longitudinal patterns in insulin and sulfonylurea treatment from before pregnancy to the second trimester. Horizontal flows show the proportion of users of a treatment strategy, alone or in combination, as use changes. “Diabetes treatment” references any diabetes medications, alone or in combination, not including insulin or sulfonylurea. Panel A shows proportions among 27,632 Medicaid women (2000 – 2014) and panel B shows proportions among 4,251 privately insured women (2004 – 2014).
